# Trophic innovations fuel reef fish diversification

**DOI:** 10.1038/s41467-020-16498-w

**Published:** 2020-05-29

**Authors:** Alexandre C. Siqueira, Renato A. Morais, David R. Bellwood, Peter F. Cowman

**Affiliations:** 1 ARC Centre of Excellence for Coral Reef Studies, James Cook University, Townsville, QLD 4811 Australia; 2 College of Science and Engineering, James Cook University, Townsville, QLD 4811 Australia

**Keywords:** Biogeography, Evolutionary ecology, Coevolution, Phylogenetics, Speciation

## Abstract

Reef fishes are an exceptionally speciose vertebrate assemblage, yet the main drivers of their diversification remain unclear. It has been suggested that Miocene reef rearrangements promoted opportunities for lineage diversification, however, the specific mechanisms are not well understood. Here, we assemble near-complete reef fish phylogenies to assess the importance of ecological and geographical factors in explaining lineage origination patterns. We reveal that reef fish diversification is strongly associated with species’ trophic identity and body size. Large-bodied herbivorous fishes outpace all other trophic groups in recent diversification rates, a pattern that is consistent through time. Additionally, we show that omnivory acts as an intermediate evolutionary step between higher and lower trophic levels, while planktivory represents a common transition destination. Overall, these results suggest that Miocene changes in reef configurations were likely driven by, and subsequently promoted, trophic innovations. This highlights trophic evolution as a key element in enhancing reef fish diversification.

## Abstract (web summary)

Both geography and ecology can drive the origins of new species. Siqueira et al. show how geological changes in the structure of Miocene reefs and the concurrent evolution of new feeding strategies combine to explain why coral reefs contain such a diversity of fish species.

## Introduction

The heterogeneity in rates of species formation across the tree of life is a widely recognized macroevolutionary pattern^1–3^. As a product of speciation and extinction rates, diversification varies through time^4^ and among lineages^1^, being ultimately influenced by both biotic and abiotic factors^5^. Consequently, understanding how biotic and abiotic factors interact in space and time is paramount in explaining underlying patterns of species diversification. For instance, recent studies have shown that distinct diversification trajectories within vertebrate groups can be explained by geographical^6,7^ and species-specific biological traits^8,9^. Although these studies provided important insights into individual drivers of vertebrate radiations, disentangling the simultaneous influence of multiple factors on rate heterogeneities is still challenging. This will require a comprehensive approach with methods that can account for multi-order interactions among the underlying drivers of species diversification.

Coral reefs constitute an excellent system for applying such broad macroevolutionary approaches, given their status as cradles for biodiversity^10^. Particularly important within these high-diversity systems, fishes represent key energetic conduits, taking part in a large proportion of the recognized biotic interactions. This ecological diversity is also reflected in taxonomic terms, with reef-associated fishes being one of the most speciose vertebrate assemblages in the world^11^. Although it has been shown that the association with reefs was an important promoter of fish cladogenesis^12^, the specific mechanisms driving this diversification are not yet fully understood. Historical and geological processes have clearly influenced global distribution patterns of reef fishes at large temporal scales^13–18^, with Miocene (23–5.3 million years ago [Ma]) changes in reef configuration being posited as one of the most important drivers of lineage expansion^19^. However, it is still unknown whether this change in the pace of lineage formation in the Miocene occurred under the influence of biotic or predominantly abiotic factors.

As recently suggested^19^, the drivers of reef fish diversification in the Miocene seem to have involved a complex mix of history and ecology. On the historical side, this epoch was marked by major geomorphological changes that reshaped marine biogeography with the formation of the Indo-Australian-Archipelago (IAA) marine biodiversity hotspot^14^. This process was likely associated with rapid diversification of reef fish lineages^12^, given the extensive opportunities for vicariance and range expansion provided by the geographical complexity of the IAA. On the ecological side, key trophic innovations (i.e., evolutionary novelties that granted access to previously unexplored resources^20^) in reef-associated fishes have fundamentally altered the nature of Miocene reefs^19^. While major reef fish trophic groups were already represented in the Eocene (56–33.9 Ma), specialized morphologies associated with the exploitation of detrital and corallivore trophic pathways, for example, only arose in the Miocene^19,21–24^. These morphological and trophic innovations have also been linked to increased lineage origination in selected reef fish groups^21,25–28^, suggesting that trophic evolution might have had a prominent role in driving patterns of reef fish diversification. Although recent studies have independently explored these potential ecological mechanisms or geographical factors underlying reef fish diversification patterns (e.g., refs. ^16,25,29–32^), they are yet to be examined in a comparative analytical framework capable of quantifying relative support.

To fill this knowledge gap, we apply phylogenetic comparative methods in near-complete phylogenetic trees to examine the relative importance of ecological and geographical factors in explaining recent lineage origination patterns in reef fishes. More specifically, we first estimate the rates of diversification for all lineages of reef-associated fishes. Then, we apply extreme gradient boosting techniques to assess the most important variables in explaining these lineage-specific rates. Finally, we investigate the historical patterns of reef fish trophic evolution in terms of evolutionary rates and guild transitions, after having identified trophic evolution as a major driver of recent reef fish diversification. These approaches provide a complementary picture of both recent and historical rates of evolution in an important vertebrate radiation, reef fishes.

## Results

### Diversification rate heterogeneity

**Fig. 1:**
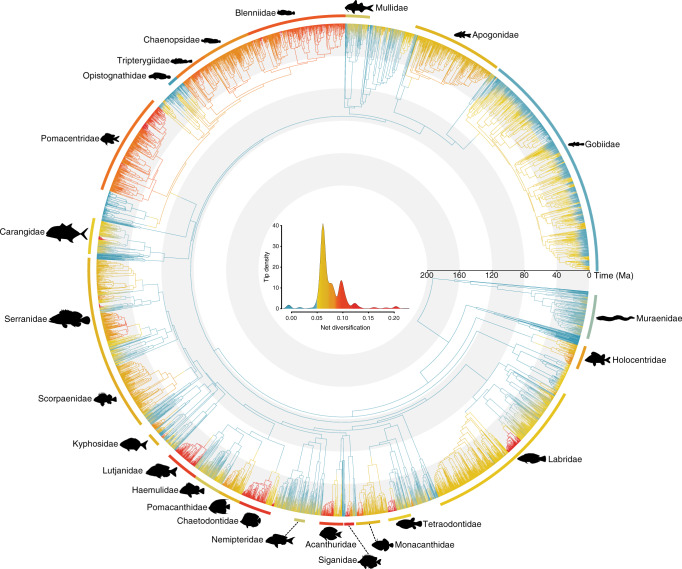
Near-complete reef fish phylogeny mapped with net diversification rates. Inset shows the overall distribution of diversification rate values for all tree tips. Blue colors represent low diversification rates, yellow intermediate, and red colors depict high diversification values. Rates were estimated through BAMM^69^. External arcs show median diversification rates estimated for some iconic reef fish families, represented by the silhouettes (sourced from Schiettekatte et al.^91^).

We found extremely heterogeneous diversification rates throughout our comprehensive reef fish trees. Net diversification rates (speciation minus extinction) varied by more than two orders of magnitude, ranging from slightly negative (−0.007 lineages Myr^−1^ [per million years]) in the genus *Megalops*, to extreme values (1.2 lineages Myr^−1^) in *Hypoplectrus*. Most extant lineages (inset, Fig. 1) and reef fish families had intermediate rates of diversification, although families such as the Siganidae, Acanthuridae, Chaetodontidae, and Lutjanidae presented a noticeably faster pace of species formation (Fig. 1). Remarkably, despite being the most speciose family on reefs, gobies presented generally low rates of diversification, particularly in the last 20 Myr (Fig. 1).

### Predictors of reef fish diversification

**Fig. 2:**
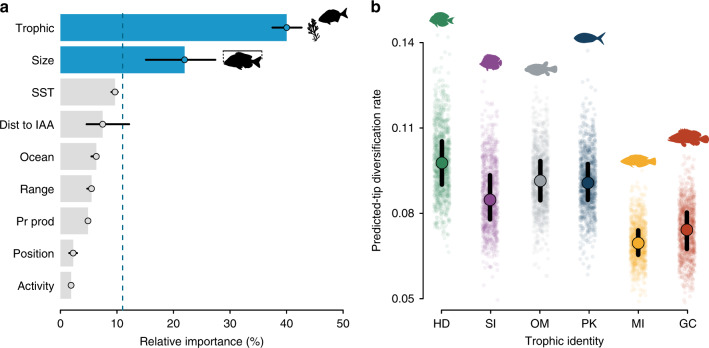
Ecological and geographical factors driving reef fish tip diversification patterns. **a** Mean relative importance (%) of explanatory variables based on an extreme gradient boosting model. Blue bars show variables above chance expectation (dashed line). Black lines represent importance quantiles (25% and 75%) derived from 1000 model bootstraps. Trophic: trophic identity; Size: maximum body length; SST: sea surface temperature; Dist to IAA: distance to the Indo-Australian-Archipelago; Ocean: oceanic basin; Range: geographic range; Pr prod: primary productivity; Position: position in the water column; Activity: circadian activity period (see Methods). **b** Predicted tip diversification rates per trophic group. In this analysis, all other continuous variables are kept at their mean values and categorical variables in the most common category. HD: herbivores/detritivores (green); SI: sessile invertivores (purple); OM: omnivores (gray); PK: planktivores (blue); MI: mobile invertivores (yellow); GC: generalized carnivores (red). Semi-transparent dots are bootstrapped predictions (*n* = 1000), with larger points representing median values with respective 25% and 75% prediction quantiles (black lines). Fish silhouettes were sourced from Schiettekatte et al.^91^. Source data are provided as a Source Data file.

Our extreme gradient boosting analysis showed that species trophic identity is the most important variable in explaining patterns in tip diversification for reef fishes. This variable had a mean relative importance of 40% in our final models (Fig. 2a). Besides trophic identity, body size was the only other variable that had a higher importance in predicting recent diversification rates than expected by chance, with a mean of 22% (Fig. 2a). All other ecological and geographical variables remained at or below the relative importance expected by chance. Overall, our model performed well, with very high prediction accuracy (mean average bias of 0.002% or 2.5%) and moderate precision (30% mean prediction variance explained).

Tip diversification rates predicted per trophic group, while keeping body size at the mean value (25 cm), were found to be highest for herbivores/detritivores (mean 0.097 [0.090–0.105; 75% prediction quantiles]) (Fig. 2b). Omnivores, planktivores, and sessile invertivores had intermediate tip diversification values (0.091 [0.084–0.098], 0.090 [0.084–0.097], 0.084 [0.077–0.093] respectively), while generalized carnivores and mobile invertivores were found to be the slowest diversifying groups (0.074 [0.067–0.080], 0.069 [0.065–0.073] respectively; Fig. 2b).

**Fig. 3:**
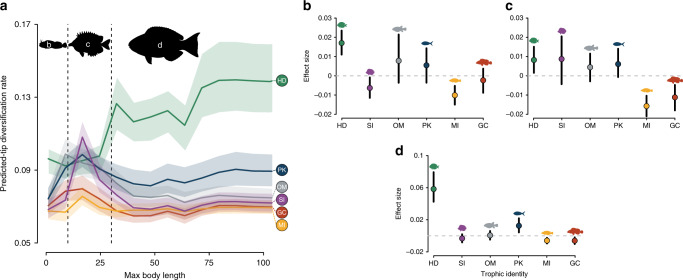
Tip diversification rates predicted for reef fish trophic groups while varying body size. **a** Predicted tip diversification rates for species of various maximum body lengths in different trophic groups, based on an extreme gradient boosting model (*n* = 1000 model bootstraps). All other variables are kept at their mean values and categorical variables (except trophic identity) in the most common category. Solid lines show median predictions per trophic group with respective prediction quantile intervals (25% and 75%). Dashed line separates size classes for which we show effect sizes per trophic group: **b** below 10 cm; **c** between 10 and 30 cm; **d** above 30 cm. In **b**–**d**, circles show the median effects (trophic group median minus global median in each size class) and black lines show 25% and 75% effect quantiles. HD: herbivores/detritivores (green); SI: sessile invertivores (purple); OM: omnivores (gray); PK: planktivores (blue); MI: mobile invertivores (yellow); GC: generalized carnivores (red). Fish silhouettes were sourced from Schiettekatte et al.^91^. Source data are provided as a Source Data file.

We also found a clear interaction between body size and trophic group (Fig. 3a). Larger HDs were predicted to have significantly higher diversification rates than smaller-bodied ones. Moreover, diversification in this group was higher than in other groups, where the rate–body size relationship flattened toward larger body-sized species (Fig. 3a). Interestingly, we found three different diversification rate regimes by dividing the results between body size classes containing a similar number of species between them. Smaller-sized species (<10 cm) were predicted to have lower tip diversification rates than larger sized ones in most trophic groups (Fig. 3a). Nevertheless, HDs were the fastest diversifying lineages in this body size class (Fig. 3b). In the intermediate size class (10–30 cm), predicted rates were higher for HDs, PKs, SIs, and OMs, when compared to the other groups (Fig. 3c). Finally, in the large body size class (>30 cm), herbivore/detritivore lineages were predicted to diversify considerably faster than any other trophic group (Fig. 3d).

Model results and predictions were consistent when we used the estimates derived from the “DR statistic” as an alternative to the BAMM estimates (see Methods), with only geographic range and temperature slightly increasing in importance (Supplementary Fig. 1). Furthermore, when we considered only the “consensus” reef fish families (i.e., universally occurring families on coral reefs, rather than “reefs” sensu lato; see Methods), we found a higher model precision (36%) with trophic identity increasing in importance (55%) when compared to other variables (Supplementary Fig. 2a). Predictions of diversification rates were similar to the main model, although rates were slightly higher in smaller to medium-sized OMs and PKs (Supplementary Fig. 2b, c). Similarly, after removing cryptobenthic fish families from the model, we found higher precision (35%) and comparable predictions, with higher rates predicted for smaller to medium OMs and PKs (Supplementary Fig. 3). This time, however, the importance of trophic group was reduced (33%) in comparison with body size (29%), suggesting that cryptobenthic fishes contribute to the trophic signal found in the main model.

### Historical patterns of trophic evolution

**Fig. 4:**
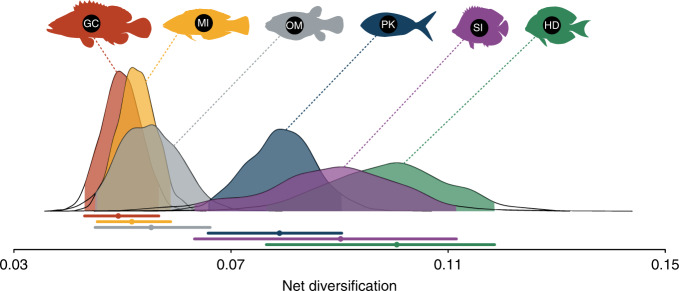
Historical net diversification rate estimates for six reef fish trophic groups. Lines below the distributions show mode values (solid circles) with respective 95% credibility intervals. Rates represent the values estimated with MuSSE for 100 trees. HD: herbivores/detritivores (green); SI: sessile invertivores (purple); OM: omnivores (gray); PK: planktivores (blue); MI: mobile invertivores (yellow); GC: generalized carnivores (red). Fish silhouettes were sourced from Schiettekatte et al.^91^. Source data is provided as a Source Data file.

Complementing the tip diversification rate results, our trophic-dependent diversification models revealed that, historically, trophic groups with more recent evolutionary origin diversified faster when compared to ancestral trophic states. HDs, SIs, and PKs had significantly higher rates of lineage formation than GCs, MIs, and OMs (Fig. 4). Apart from a few exceptions (e.g., Lutjanidae), these results are similar to those found for tip diversification rates (Fig. 2b), indicating that patterns of lineage diversification among trophic groups have been historically consistent, with recent trophic groups diversifying rapidly in the past 20 Myr (Supplementary Figs. 4 and 5). Additionally, we found that estimated speciation rates were higher than extinction rates in all groups (Supplementary Fig. 6), which resulted in positive net diversification rates for all trophic groups (Fig. 4).

**Fig. 5:**
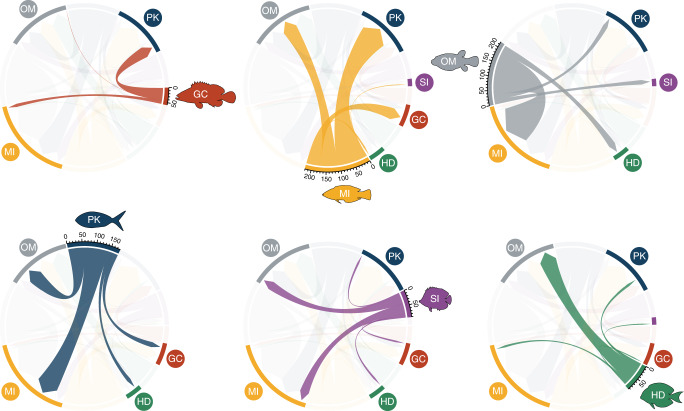
Directionality of transitions out of each reef fish trophic group. Maximum chord width represents the number of lineages averaged between 100 trees, shown in the scale. HD: herbivores/detritivores (green); SI: sessile invertivores (purple); OM: omnivores (gray); PK: planktivores (blue); MI: mobile invertivores (yellow); GC: generalized carnivores (red). Fish silhouettes were sourced from Schiettekatte et al.^91^. Source data is provided as a Source Data file.

Stochastic character mappings revealed a clear sequential pattern of transitions between trophic groups. GC and MI lineages transitioned frequently between them and to planktivory (Fig. 5). However, these groups very rarely transitioned to other trophic groups such as herbivory/detritivory or sessile invertivory. The transitions to these groups happened, almost exclusively, from omnivorous lineages (Fig. 5). Herbivore/detritivore and SI lineages occasionally transitioned back to omnivory, while PKs frequently transitioned back to mobile invertivory. Most groups exhibited frequent transitions to planktivory, making it a common trophic destination in reef fish evolution. Finally, the transitions to omnivory were predominantly made by MI lineages (Fig. 5), suggesting omnivory as an intermediate evolutionary step between lower and higher trophic levels.

## Discussion

Using near-complete phylogenies, coupled with a comprehensive ecological and geographical dataset, we identified species trophic guild and body size as major drivers of diversification in reef-associated fishes. Although the role of different types of resource use has been previously suggested as a driver of evolutionary rates in some reef fish groups^25–28,31,33^, we reveal its full potential across the complete reef fish tree of life. Through an intricate relationship with body size, the trophic identity of species was more important in predicting the pace of reef fish evolution than any other ecological or geographical factor examined. On average, herbivorous/detritivorous fish lineages diversified faster than other trophic groups. However, rate differences are amplified, rather than diminished, in large-bodied species. Alongside HDs, PKs and SIs showed faster than average historical rates of evolution, particularly in the past 20 Myr. This highlights the potential importance of new reef configurations in the Miocene^19^ in promoting trophic innovations within these guilds. Complementing the patterns of reef fish trophic evolution, we also show that planktivory and omnivory constitute key evolutionary pathways. Planktivory is the main evolutionary destination in trophic transition episodes, while omnivory appears to represent a transient state between high and low trophic levels. The major drivers of reef fish diversification and the evolutionary pathways of trophic transitions will be discussed separately below.

Trophic innovations have been previously identified as a key element in the radiation of one of the most speciose fish families on coral reefs, the Labridae^33^. Expanding the taxonomic scope, Lobato et al.^25^ suggested that ecological opportunity might have underpinned higher diversification rates in some reef fish lineages that shifted towards lower-level trophic guilds. Even though this latter study was based on a coarse trophic distinction between guilds feeding on low- and high-quality food, our results using finer trophic categories largely agree that lower-quality feeding guilds (HDs and SIs) have higher diversification rates than higher-quality feeding ones. However, in addition to these lower-quality feeding guilds, we found that planktivorous lineages also diversified disproportionally fast. This suggests that it is recently acquired trophic strategies, rather than low-quality feeding per se, that may have opened up opportunities for shifts in the pace of lineage origination. These trophic innovations in reef fishes predominantly occurred in the past 20 Myr^19,25,33^, a time that closely matches the highest diversification rates of key lineages in our study (Fig. 1) and generally across the tree of life^34^. Thus, we suggest that this increased diversification in herbivores, SIs, and PKs may be explained by ecological opportunities unveiled by fundamental changes in reef configuration occurring during the Miocene.

This geological period was marked by the rise of high-turnover reef ecosystems in which both fast-growing corals and large bioeroding fishes first appeared^19^. These fundamental changes in the dynamics of reef structure likely promoted new opportunities for trophic innovation in fishes^33^. Particularly important for the expansion of recently derived trophic groups in the Miocene appears to have been the colonization of reef flats. Evidence from present-day reefs suggests that this habitat is by far the most productive reef zone for benthic organisms, and this is reflected in their yield to grazing fishes^35,36^. However, these shallow areas of the reef are also exposed to high wave energy and fish populations may be shaped by the availability of flow and predatory refuges^37,38^. Thus, these habitats appear to offer potential benefits but they also present substantial challenges for most fishes^37,38^. In evolutionary terms, although some typical herbivorous reef fish families arose and expanded in the Paleocene–Eocene (66–33.9 Ma)^18^, it was not until the Miocene that they acquired necessary body and fin morphologies to move into this challenging reef zone^23,24^. This apparently simple move may have driven profound trophodynamic changes in shallow reefs^36^, which might help explain our results.

There are three key components to this explanation. First, the colonization of the productive reef flats probably allowed herbivorous fishes to expand their population sizes^36^. Second, the intense grazing pressure promoted by large herbivorous populations may have facilitated the expansion of corals in shallow waters, by altering the coral–algal competitive balance^19^. This is supported by the paleontological evidence, which suggests that, despite some peripheral scleractinian reef formation in the Eocene^39^, the rise of modern scleractinian-dominated reefs only took place in the late Miocene^39–41^. Finally, once corals dominated shallow waters, they had the capacity to promote the expansion of sessile invertivorous and planktivorous lineages. In the case of SIs, this expansion was likely related to more opportunities for resource exploitation, given the general increase in the availability of both shelter and the abundance of organisms exploited as food sources^19^. In PKs, the expansion was potentially linked to the shelter provided by topographical complexity against predators and water flow in highly productive and highly hydrodynamic shallow reef environments^42^. Although intense hydrodynamics might offer a constant flow of planktonic resources, without the refuge provided by corals these shallow reef habitats would probably be uninhabitable for many planktivorous species^43^.

This hypothesized scenario provides not only a logical explanation for the observed diversification rates among trophic groups, but it also helps elucidating the patterns found for large body-sized herbivores. To meet metabolic demands, herbivorous fishes have to maintain higher feeding rates when compared to other trophic groups^44^. However, by doing this, these fishes become more exposed to predation^45^. As body size is a major determinant of predation risk in reef fishes^46^, being large may provide herbivorous fishes with a size refuge from predation. Consequently, the colonization of the reef flats in the Miocene might have been particularly beneficial for large-bodied herbivorous fishes. This is because they were free to maintain high grazing rates on highly productive reef flats, while avoiding the typically high predation pressure in these habitats^36^. Thus, predation could have been a key component in driving differences in the diversification rate of small–medium and large herbivorous fishes. Although speculative, these ideas provide a fertile ground for future studies willing to compare different models of size evolution in herbivorous reef fishes.

In addition to the predation effect, body size has also been shown to correlate positively with geographical range in reef fishes^47^, which highlights the importance of this trait for species’ long-distance colonization capabilities. Although this might promote genetic connectivity in ecological time scales, in evolutionary time scales it might also increase the chances of vicariance, given the variability in effectiveness of marine biogeographical barriers through time^13,48,49^ and the likelihood of fragmentation of previously contiguous populations. While body size can be considered a “universal trait” related to multiple biological processes^50^, the key element here might be related to use of shallow reef flat habitats. It appears that the remarkably higher diversification rates found for large-bodied HDs (Fig. 2f) was probably related to a combination of higher population sizes, driven by colonization of highly productive reef flats and low mortality, coupled with long-distance dispersal potential within these lineages.

For most trophic identities, our recent and historical approaches provided similar results. However, we found a decoupling between high tip and low historical rates estimated for OMs (Figs. 2b, 4). This suggests that omnivorous lineages might have experienced only limited rates of origination in the past, counterbalancing recent expansions. Alternatively, this decoupling might be related to the transient nature of omnivory through time, which would result in short-lived evolutionary lineages within that trophic group. Interestingly, omnivorous lineages have previously been shown to be the slowest evolving groups in both mammals^8^ and birds^9^. In the latter group, extinction rates were estimated to be even higher than speciation, leading the authors to flag omnivory as a macroevolutionary sink. Although this was not the case for reef fishes in both small^31^ and large taxonomic scales, low historical rates of diversification in omnivorous lineages seem to be a common pattern in vertebrate evolution.

Herbivores have also been found to be the fastest diversifying lineages in many disparate vertebrate and invertebrate taxa (e.g., refs. ^8,9,51^), suggesting that animal trophic evolution might follow common rules. However, to our knowledge, this is the first time that the synergistic effects of species body size and trophic identity have been considered simultaneously when exploring diversification patterns in vertebrates. Body size is regarded as an important component of organismal evolution, given its influence on metabolic rates^52^ and generation times^53^. Thus, our results showing lower diversification rates for smaller-bodied species seem counterintuitive considering evolutionary theories. For example, small cryptobenthic fishes contribute to a large proportion of the species richness found on coral reefs^54^, and their high population turnover and low connectivity should promote faster rates of diversification^55^. Yet, our results show that gobies, for instance, might be among the slowest evolving families on coral reefs. We propose two possible explanations for these seemingly counterintuitive results. First, although the fast life history of cryptobenthic fishes should be reflected in rapid diversification, some groups might be experiencing high rates of extinction. Unfortunately, estimating extinction rates from phylogenetic trees can be problematic^56^, making the test of this hypothesis difficult without a good fossil record. Second, our rates of diversification for cryptobenthic fishes might be underestimated due to taxonomic sampling (judging by the rate of species descriptions for these groups and the expected number of undescribed species^54^). While plausible, when we controlled for this effect by removing key cryptobenthic families^54^, our trophic results remained practically unchanged and we still found slightly lower diversification rates for smaller-bodied species (Supplementary Fig. 3). Nevertheless, this might be an important topic for further investigation in attempts to clarify the relationship between diversification rates and body size in reef fishes.

In terms of evolutionary trophic pathways, transitions to planktivory have long been recognized as one of the most recurrent patterns in reef fish evolution^57^, with examples occurring consistently across a broad range of families^58^. However, our study represents the first effort to quantify this pattern using a large-scale phylogenetic framework. It is also recognized that these shifts are associated with specific morphological and behavioral changes related to food acquisition (e.g., refs. ^59,60^). Despite being unusual in other trophic identities, these morphological modifications (e.g., slender fusiform bodies and deeply forked caudal fins^58^) associated with planktivory seem to arise frequently, no matter the trophic group of the originating lineage (Fig. 5). Not surprisingly, reef fish PKs nested within groups with more generalized morphologies are often described as separate genera due to differences in body and caudal shape, despite only representing a shift to a feeding mode higher in the water column^58^. One hypothesis that may explain this pattern is that recurrent transitions to planktivory in adult stages should be an easier evolutionary step compared to other trophic transitions simply because most reef fishes have already been planktivorous in early life stages^57^.

In other recently derived trophic groups, however, transitions occur almost exclusively from omnivorous lineages (Fig. 4), a finding that matches previously described patterns in the Labridae and Pomacentridae^31^. HDs and SIs have numerous specific morphological, physiological, and behavioral attributes (e.g., refs. ^61,62^) that are unlikely to be simply acquired in evolutionary terms. Not coincidently, these trophic identities represent the most taxonomically restricted groups of reef fishes. Thus, as suggested for selected reef fish families^31^, the pathway to transition within these trophic groups appears to involve an intermediate generalist stage in which lineages have not yet fully developed the biological traits related to the exploration of specific resources. Interestingly, omnivorous reef fishes have been shown to have very slow rates of morphological evolution^29^. Alongside our results, this suggests that omnivory might not be an evolutionarily stable trophic strategy; rather, it may represent a transitional stage between reef fish trophic groups.

Finally, it is important to make some model considerations. While it has recently been demonstrated that deep temporal trends in speciation and extinction rates cannot be reliably identified from phylogenies containing extant species only^63^, our study is unlikely to suffer from this issue. This is because our model relies on estimates of very recent diversification rates (tip rates), which have been shown to be relatively robust to the issues of parameter non-identifiability^63^. Furthermore, considering the extreme heterogeneity in diversification rates found in reef fishes (Fig. 1), and the multitude of other potential explanatory variables that were not included in our model, an average of 30% of explained variance can be regarded as a good performance for an intuitively simple model such as ours. Reef fishes have extraordinarily diverse life and evolutionary histories; therefore, it is remarkable that a coarse trophic distinction and maximum species body size alone can explain almost one-third of the variability in diversification rates. It is hard to conceive another single factor that could have a higher explanatory power than the ones found herein. Additionally, when we considered only the “consensus” reef fish families, our model explained an even higher proportion of the variability (36%), with trophic group increasing considerably in importance (55%). This suggests that our diversification rate results were most strongly associated with the history of coral reefs and not with peripheral environments that also support “reef-associated” fish species.

In conclusion, trophic innovations are closely tied to evolutionary rate shifts in reef-associated fishes. Relative to all other trophic groups, herbivorous fishes have sustained remarkably fast diversification rates, a pattern that is particularly pronounced in large body-sized species. This combination is likely related to their ecological success after colonizing the productive reef flat during the Miocene. Acting through an evolutionary cascade, the colonization of this zone appears to have triggered profound changes in reef configuration, which in turn underpinned critical trophodynamic shifts and the diversification of other trophic groups. These cascading effects were likely mediated by recurrent transitions between guilds. While planktivory represents a common evolutionary route in reef fish evolution, omnivory might have provided the critical transitional link between higher and lower trophic levels. Overall, our results suggest the existence of a mechanistic basis underpinning the role of trophic evolution in determining macroevolutionary patterns in reef fishes.

## Methods

### Reef fish phylogeny

We built a comprehensive phylogeny of reef fish species, based on a recently published chronogram of ray-finned fishes^7^. This chronogram was constructed using a 27-gene alignment for 11,638 actinopterygian species and was time calibrated using a comprehensive dataset of fossil occurrences. We downloaded the Rabosky et al.^7^ chronogram from fishtreeoflife.org. Then we used the “ape”^64^ R package to prune down the tree, restricting it to reef-associated taxa. Since the definition of what constitutes a reef fish is a contentious subject^13^, we used a systematic approach in selecting the species to be kept in the tree. Starting from the full list of fish families with reef-associated species from Bellwood and Wainwright^13^, we used the “rfishbase”^65^ R package to access the list of all valid species within each of those families and then calculate the proportion that were classified as reef associated. Finally, we selected families with more than 20% of reef-associated species and kept them in the tree. The final pruned chronogram contained 2585 species in 65 families.

This time-calibrated pruned tree was subsequently used as a backbone for the imputation of all missing species within each of the selected families. To do this, we generated a list of all valid reef-associated species belonging to the selected families based on FishBase^66^ and the Eschemeyer’s Catalog of Fishes^67^. We then assigned taxonomic ranks to all species present in the list using the same online datasets, but also using information from the backbone tree to better define monophyletic groups. With this taxonomic dataset, we applied the TACT stochastic polytomy resolution algorithm^68^, which uses birth–death models to calculate diversification rates for taxonomic ranks and inputs missing species within the most restrictive ranks according to the respective calculated rate. This method has the advantage of estimating local diversification rates, as opposed to global rates, being more suitable for large phylogenies with heterogeneous rate regimes^7^. Although our approach is very similar to the one implemented by Rabosky et al.^7^ to build a near-complete tree, we used more restrictive taxonomic ranks in an attempt to narrow down the placement of missing species. In most cases, missing species were placed within their respective genera or, at least, within their respective subfamilies where available. Finally, to account for stochastic variability in the placement of missing species within genera/subfamily, we generated a distribution of 100 near-complete reef fish trees, each containing 6257 tips.

### Diversification rates

To estimate diversification rates within our phylogenies, we used the program BAMM 2.5.0^69^. This program uses a Bayesian framework and a reversible-jump Markov Chain Monte Carlo (rjMCMC) process to find distinct diversification regimes within a phylogeny and estimate lineage-specific speciation and extinction rates. For each of our trees, we ran time-variable models for 30 million generations using default operators and priors generated through the “BAMMtools”^70^ R package. To facilitate convergence, we set a prior expectation of 100 diversification regime shifts. Since we were using near-complete trees, we set the globalSamplingFraction parameter to one. At the end of each run, we removed the initial 10% of the samples as burn-in and assessed convergence through the effective sample sizes using the “coda”^71^ R package. After running BAMM independently in each of our 100 trees, we combined their results by assessing the median estimated tip diversification rates.

Although concerns related to BAMM have been raised (e.g., refs. ^72,73^), they have been largely addressed in subsequent studies and program refinements (e.g., refs. ^74,75^). The current program, therefore, remains a robust framework for estimating diversification rates in large phylogenetic trees. Recently, another framework has been proposed (ClaDS^3^), providing model improvements in terms of lineage-specific rate estimates. Although this model represents a very strong alternative to BAMM, its implementation is still computationally very intensive, making analyses in large phylogenies such as ours impractical. Therefore, to be able to use another method to cross-validate our main BAMM analysis, we applied the “DR statistic”^6^ in our near-complete trees. Although this method is mainly focused on speciation, rather than diversification rates^76^, it is a very useful metric to study speciation rate dynamics alongside BAMM^76^. We applied this method in our 100 trees and assessed the median lineage-specific speciation rates. The median BAMM and the DR tip estimates were then used independently as the response variables in our predictive model (see Predicting diversification rates in Methods section). Finally, since we focused on patterns of recent (tip) diversification rates, our estimates are unlikely to be influenced by the recently described issues of parameter non-identifiability^63^ in extant species phylogenies.

### Explanatory variables

To assess the main drivers of diversification in reef fishes, we generated a dataset with potential explanatory variables. These variables consisted of a set of species’ ecological traits and geographical factors hypothesized to influence the pace of reef fish lineage formation. We used information from the literature, online datasets, and expert assessments^58,66,67,77,78^ to classify species according to a continuous trait reflecting body size (maximum body length), and three categorical traits related to species’ ecologies (trophic identity, activity pattern, and position in the water column). All the body length data available for our studied species was downloaded from FishBase through “rfishbase”^65^. For the trophic identity, we grouped species into six major categories related to their diets in the adult life stages: GCs, MIs, OMs, PKs, SIs, and HDs. These categories are related to previously defined dietary groups for reef fishes^77^; however, we merged the herbivores/macroalgivores category with the general herbivores/detritivores group. This was done to avoid biases in the predictive and the trait-dependent diversification models, given the very small sample size of macroalgivores in our dataset. Our classification considered the most common diets described for each species regardless of potential geographical variation. We also split species between diurnal, nocturnal, or both^77^, according to their circadian pattern of activity. Lastly, we used the vertical position where fishes are commonly found in the water column as a proxy for their degree of association with the reef matrix, so we classified species as benthic, benthopelagic, and pelagic^77^.

To classify species according to geographical variables, we downloaded the occurrence-based dataset from Rabosky et al.^7,79^. This dataset consists of global presence–absence records of fishes in 300 × 300 km^2^ resolution grids, originally sourced from four online biodiversity information systems (GBIF, OBIS, Fishnet2, and VertNet)^7^. From this presence–absence data, we filtered those species that were present in our trees and we calculated their geographical range by summing the number of occupied cells. Additionally, we classified species according to their presence in each major oceanic basin (Atlantic, Indo-Pacific, or both), and we calculated the absolute latitude of the centroid of their geographical distribution. By combining the absolute latitude value with the longitudinal centroid of each species, we calculated the distance between that centroid and a central point in the IAA (Lat 0; Long +121). These variables were added to the model (see Predicting diversification rates in Methods section) to assess predictions related to the influence of biogeography into reef fish diversification rates^12,19^.

In addition to the presence–absence dataset, we also downloaded the supplementary data from Rabosky et al.^7,79^ that contained environmental variables per grid cell. With these data, we accessed the mean sea surface temperature (SST) and the mean primary productivity (Pr prod) at the centroid grid of each species. Since tropical reef fish lineages have been found to sustain higher net diversification rates^30^, we used these variables to assess if this might be associated with higher temperatures or energy availability. Our complete dataset containing species’ ecological traits (body size, trophic identity, activity, and position) and geographical variables (geographic range, oceanic basin, distance to IAA, mean SST, and mean Pr prod) had a total of 4875 species.

### Predicting diversification rates

To evaluate the importance of each ecological and geographical variables in predicting reef fish diversification rates, we used the Gradient Boosted Regression Tree method XGBoost^80^. This machine learning technique represents a state-of-the-art method for modelling complex nonlinear relationships^81^. It has advantages over other modelling techniques because it automatically handles multi-order interactions among predictors, it does not require prior data transformation or outlier exclusion^81^, and it provides fast and accurate predictions^80^. We used the “xgboost”^82^ R package to build our predictive model. Before running the predictive model, we performed two tuning steps to obtain the combination of parameters (learning rate, maximum tree depth, gamma, and subsampling rate) that would result in the minimum root mean square error (rmse). In the first tuning step, we fit models with a range of predefined parameter combinations that were varied systematically to assess which would provide the minimum rmse. In the second tuning step, we refit 1000 models by randomly sampling parameters from a uniform distribution with upper and lower bounds defined as values from the best parameter combination of the first step plus or minus 10%. The parameter combination with the minimum rmse from the second tuning step was then used in the final predictive model. Both tuning steps and the final predictive model were fitted using a gamma distribution for the median tip diversification rates resulting from our BAMM analysis as the response variable.

We used a cross-validation procedure to assess the model’s accuracy and precision in predicting diversification rates. To do that, we divided our dataset into training and testing parts by randomly subsetting 80% and 20% of the datapoints, respectively. We used the training dataset to refit the final model and assess the coefficients of prediction. These coefficients were then used to predict the tip diversification rates in the testing dataset. Accuracy was calculated as the average bias by subtracting each predicted tip diversification from its actual value in the training dataset. Precision was assessed using the *R*^2^ of a linear model fitted between the measured and predicted diversification rates. These cross-validation tests were performed 1000 times to assess the mean accuracy and precision values.

We ran the predictions for all levels of the categorical variables, and for a range of values spanning the minimum and maximum measured continuous variables. These predictions were bootstrapped for 1000 iterations to assess the relative importance of each explanatory variable. Finally, we did another 1000 bootstrap iterations of the final predictive model varying only trophic group and maximum body length (the most important variables; see Results), while keeping all the other continuous variables in their mean values and the categorical variables in their most common category.

All of these steps were replicated using the “DR statistic” results as the response variable. Moreover, two model sensitivity analyses were performed. First, we ran the *xgboost* analysis selecting only the reef fish families considered “consensus” families^13^ (i.e., Acanthuridae, Apogonidae, Blenniidae, Carangidae, Chaetodontidae, Holocentridae, Labridae, Mullidae, Pomacanthidae, Pomacentridae, Serranidae). Second, we ran the predictive model excluding the families defined by Brandl et al.^54^ as cryptobenthic reef fishes. With the first analysis, we intended to eliminate potential issues of defining what constitutes reef fishes. In the second analysis, we wanted to exclude the potential taxonomic bias associated with smaller body-sized species, that is, we expect more undescribed cryptobenthic species than larger-bodied ones.

### Trait-dependent diversification

After detecting trophic identity as the main explanatory variable for recent (tip) patterns of lineage diversification in reef fishes (see Results), we explored the historical patterns of trophic evolution using the whole structure of the phylogenetic trees. This was achieved by building multistate speciation and extinction (MuSSE^83^) models for the classified trophic groups. Our two sets of diversification analyses differ in the sense that the first (BAMM) was used to estimate rates independently of trait evolution, whereas the second (MuSSE) was specifically used to investigate trait-dependent patterns of diversification. These trait-dependent diversification models allow the analysis of character state evolution coupled with changes in speciation and extinction rates.

For each of our reef fish trees, we estimated the parameters (speciation, extinction, and transition rates) associated with each trophic group using an unconstrained MuSSE model with the maximum-likelihood function of the “diversitree”^83^ R package. Subsequentially, we used the resulting maximum-likelihood coefficients to apply the Bayesian framework of “diversitree” and sample the posterior probability distribution of parameters. We ran the MCMC chain for 2000 generations with exponential priors from a preliminary run of 100 generations. After each run, we excluded 10% of the samples as burn-in and assessed convergence using the effective sample sizes. Finally, we combined the post burn-in samples from all trees and calculated net diversification rates by subtracting extinction rates from speciation rates.

Issues related to the model selection procedure of trait-dependent diversification models have been previously identified^84^; however, they are unlikely to affect our analysis. This is because we did not use MuSSE to perform model selection and thus imply that trophic group is the only trait affecting reef fish diversification. Based on our BAMM results (Fig. 1), we know that the diversification regime in the full reef fish tree is highly heterogeneous and it was unlikely influenced by only one trait. Because we detected trophic identity as an important variable for explaining tip diversification rate variability in reef fishes, we used this method exclusively to explore full-tree patterns. Thus, our trait-dependent analysis should be viewed as a complementary resource to the results found with the trait-independent one (BAMM). As a way to alleviate potential issues with the trait-dependent analysis, we applied the HiSSE method^85^ by splitting our trophic categories between ancestral (GCs, MIs, and OMs) and more recently derived (HDs, SIs, and PKs) groups. Using the HiSSE framework, we built an unconstrained model that considered rates to be different between analyzed character states (trophic group) with one hidden diversification regime per state, and compared it to a model in which rates were constrained between states but different from the hidden diversification regime. Results from this HiSSE analysis supported our MuSSE results (see “Results”) and estimated higher diversification rates for the recently derived trophic groups compared to the ancestral ones (Supplementary Table 1).

### Trophic transitions

We quantified the transitions between classified trophic groups by using stochastic character mappings^86^. Considering that rate heterogeneity can affect the results of ancestral state reconstructions^87^, we used the results of our trait-dependent diversification model (MuSSE) to perform this analysis. For each of our near-complete trees, we simulated 10 stochastic maps using a modified version of the make.simmap function from the “phytools”^88^ R package. We customized the aforementioned function to use the transition rates and the ancestral state reconstruction results derived from the original MuSSE model (asr.marginal function in “diversitree”) as inputs for the stochastic mappings. The combined results of all stochastic maps were summarized to assess the mean number of transitions per trophic group. These estimates were then used to plot chord diagrams representing the directionality of transitions using the “circlize”^89^ R package.

### Reporting summary

Further information on experimental design is available in the Nature Research Reporting Summary linked to this paper.

## Supplementary information

Supplementary Information

Peer Review

Reporting Summary

## Data Availability

The datasets generated during and/or analyzed during the current study are available at the James Cook University’s Tropical Data Hub repository (10.25903/5e9659dbca234)^90^. There are no restrictions on data availability. The phylogeny used as backbone was downloaded from The Fish Tree of Life (https://fishtreeoflife.org). Publicly available datasets used in the study include: FishBase (http://www.fishbase.org/search.php), Eschemeyer’s Catalog of Fishes (http://researcharchive.calacademy.org/research/ichthyology/catalog/fishcatmain.asp), and the Dryad repository of Rabosky et al.^79^ (10.5061/dryad.fc71cp4). The source data underlying Figs. 2a–b, 3a–d, 4, and 5 and Supplementary Figs. 1a–f, 2a–f, 3a–f and 6 are provided as a Source Data file.

## Source data

Source Data

## Code availability

The codes used during the current study are available at the James Cook University’s Tropical Data Hub repository (10.25903/5e9659dbca234)^90^.
