# Supplementary material for: Trophic innovations fuel reef fish diversification
Source: Nat Commun. 2020 May 29;11:2669. doi: 10.1038/s41467-020-16498-w (PMC7260216; doi:10.1038/s41467-020-16498-w)
Supplement: Supplementary file 1 — Supplementary Information [file 41467_2020_16498_MOESM1_ESM.pdf]

***Supplementary information for*:**

Trophic innovations fuel reef fish diversification

Nature communications

Alexandre C. Siqueira[1]*; Renato A. Morais[1,2]; David R. Bellwood[1,2]; Peter F. Cowman[1]

[1] ARC Centre of Excellence for Coral Reef Studies, James Cook University, Townsville, QLD 4811, Australia.

[2] College of Science and Engineering, James Cook University, Townsville, QLD 4811, Australia.

[*] Author for Correspondence: Alexandre C. Siqueira, alexandre.siqueira@my.jcu.edu.au

Supplementary figures

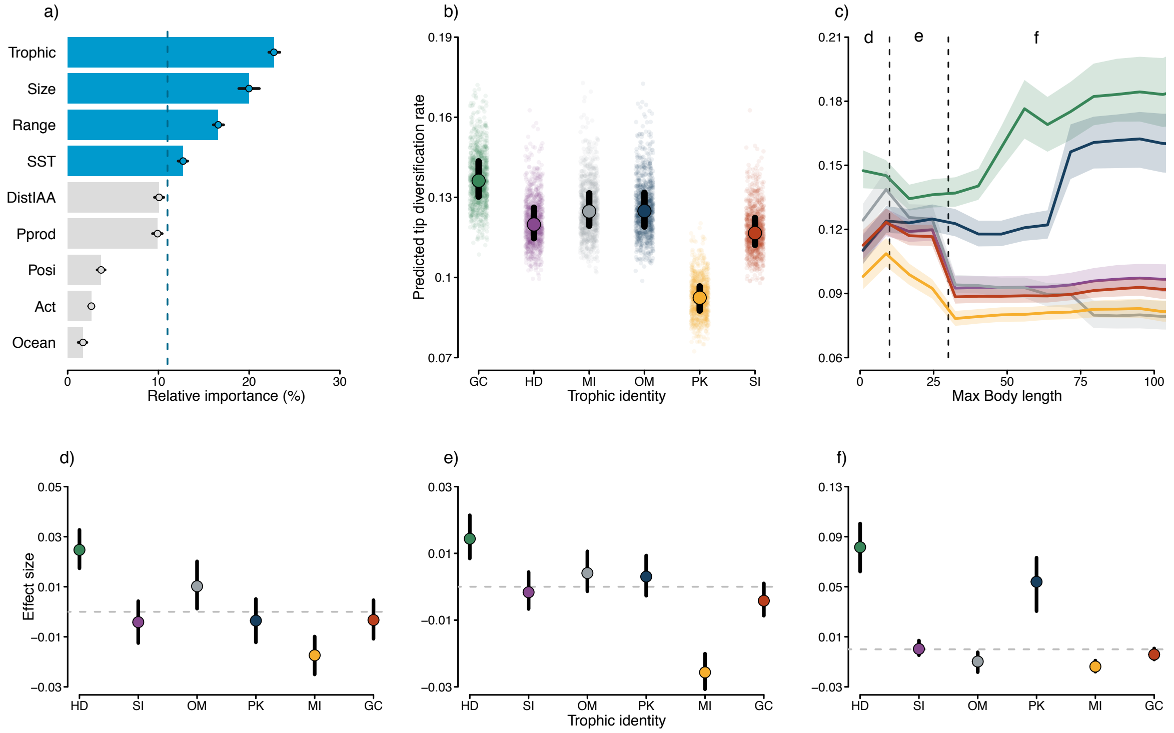

Supplementary Figure 1. Relative importance of ecological and geographical factors in driving reef fish tip diversification rate patterns, based on an extreme gradient boosting model using the 'DR statistic' estimates as response variable. (a) Mean relative importance (%) of explanatory variables. Blue bars show variables above chance expectation (dashed line). Black lines represent importance quantiles (25% and 75%) derived from 1000 model bootstraps. Trophic: trophic identity; Size: maximum body length; SST: sea surface temperature; DistIAA: distance to the Indo-Australian-Archipelago; Ocean: oceanic basin; Range: geographic range; PProd: primary productivity; Posi: position in the water column; Act: circadian activity period (see methods). (b) Predicted tip diversification rates per trophic group. In this analysis, all other continuous variables are kept at their mean values and categorical variables in the most common category. Semi-transparent dots are bootstrapped predictions (n = 1000), with larger points representing median values with respective 25% and 75% prediction quantiles (black lines). (c) Predicted tip diversification rates for species of various maximum body lengths in different trophic groups, based on an extreme gradient boosting model (n = 1000 model bootstraps). All other variables are kept at their mean values and categorical variables (except trophic identity) in the most common category. Solid lines show median predictions per trophic group with respective prediction quantile intervals (25% and 75%). Dashed line separates size classes for which we show effect sizes per trophic group: (d) below 10 cm; (e) between 10 and 30 cm; (f) above 30 cm. In d-f, circles show the median effects (trophic group median minus global median in each size class) and black lines show 25% and 75% effect quantiles. HD: herbivores/detritivores (green); SI: sessile invertivores (purple); OM: omnivores (grey); PK: planktivores (blue); MI: mobile invertivores (yellow); GC: generalized carnivores (red). Source data are provided as a Source Data file.

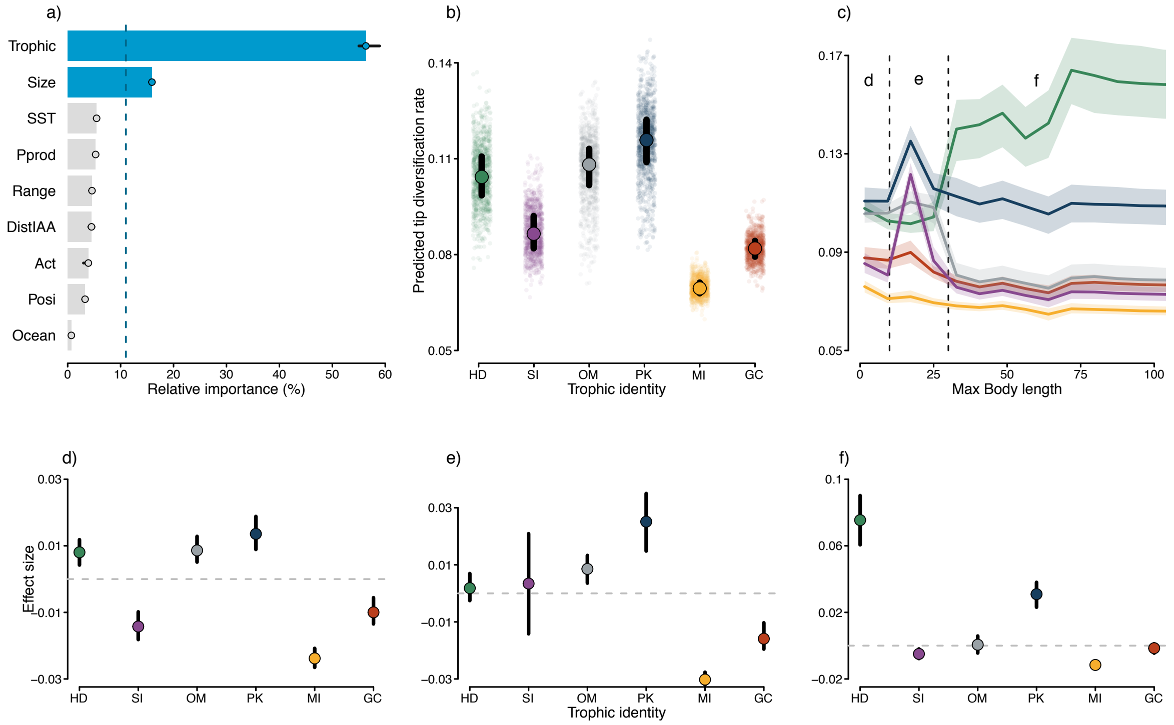

Supplementary Figure 2. Relative importance of ecological and geographical factors in driving reef fish tip diversification rate patterns, based on an extreme gradient boosting model using only the 'consensus' reef fish families. (a) Mean relative importance (%) of explanatory variables. Blue bars show variables above chance expectation (dashed line). Black lines represent importance quantiles (25% and 75%) derived from 1000 model bootstraps. Trophic: trophic identity; Size: maximum body length; SST: sea surface temperature; DistIAA: distance to the Indo-Australian-Archipelago; Ocean: oceanic basin; Range: geographic range; PProd: primary productivity; Posi: position in the water column; Act: circadian activity period (see methods). (b) Predicted tip diversification rates per trophic group. In this analysis, all other continuous variables are kept at their mean values and categorical variables in the most common category. Semi-transparent dots are bootstrapped predictions (n = 1000), with larger points representing median values with respective 25% and 75% prediction quantiles (black lines). (c) Predicted tip diversification rates for species of various maximum body lengths in different trophic groups, based on an extreme gradient boosting model (n = 1000 model bootstraps). All other variables are kept at their mean values and categorical variables (except trophic identity) in the most common category. Solid lines show median predictions per trophic group with respective prediction quantile intervals (25% and 75%). Dashed line separates size classes for which we show effect sizes per trophic group: (d) below 10 cm; (e) between 10 and 30 cm; (f) above 30 cm. In d-f, circles show the median effects (trophic group median minus global median in each size class) and black lines show 25% and 75% effect quantiles. HD: herbivores/detritivores (green); SI: sessile invertivores (purple); OM: omnivores (grey); PK: planktivores (blue); MI: mobile invertivores (yellow); GC: generalized carnivores (red). Source data are provided as a Source Data file.

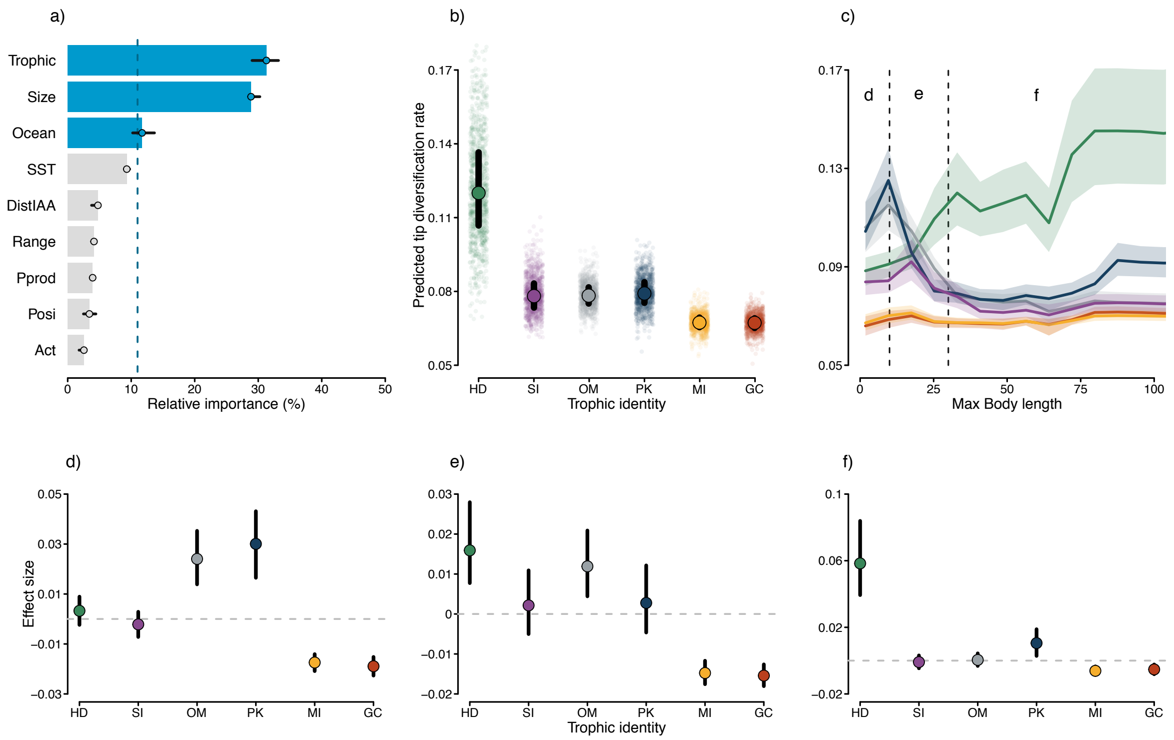

Supplementary Figure 3. Relative importance of ecological and geographical factors in driving reef fish tip diversification rate patterns, based on an extreme gradient boosting model excluding cryptobenthic families. (a) Mean relative importance (%) of explanatory variables. Blue bars show variables above chance expectation (dashed line). Black lines represent importance quantiles (25% and 75%) derived from 1000 model bootstraps. Trophic: trophic identity; Size: maximum body length; SST: sea surface temperature; DistIAA: distance to the Indo-Australian-Archipelago; Ocean: oceanic basin; Range: geographic range; PProd: primary productivity; Posi: position in the water column; Act: circadian activity period (see methods). (b) Predicted tip diversification rates per trophic group. In this analysis, all other continuous variables are kept at their mean values and categorical variables in the most common category. Semi-transparent dots are bootstrapped predictions (n = 1000), with larger points representing median values with respective 25% and 75% prediction quantiles (black lines). (c) Predicted tip diversification rates for species of various maximum body lengths in different trophic groups, based on an extreme gradient boosting model (n = 1000 model bootstraps). All other variables are kept at their mean values and categorical variables (except trophic identity) in the most common category. Solid lines show median predictions per trophic group with respective prediction quantile intervals (25% and 75%). Dashed line separates size classes for which we show effect sizes per trophic group: (d) below 10 cm; (e) between 10 and 30 cm; (f) above 30 cm. In d-f, circles show the median effects (trophic group median minus global median in each size class) and black lines show 25% and 75% effect quantiles. HD: herbivores/detritivores (green); SI: sessile invertivores (purple); OM: omnivores (grey); PK: planktivores (blue); MI: mobile invertivores (yellow); GC: generalized carnivores (red). Source data are provided as a Source Data file.

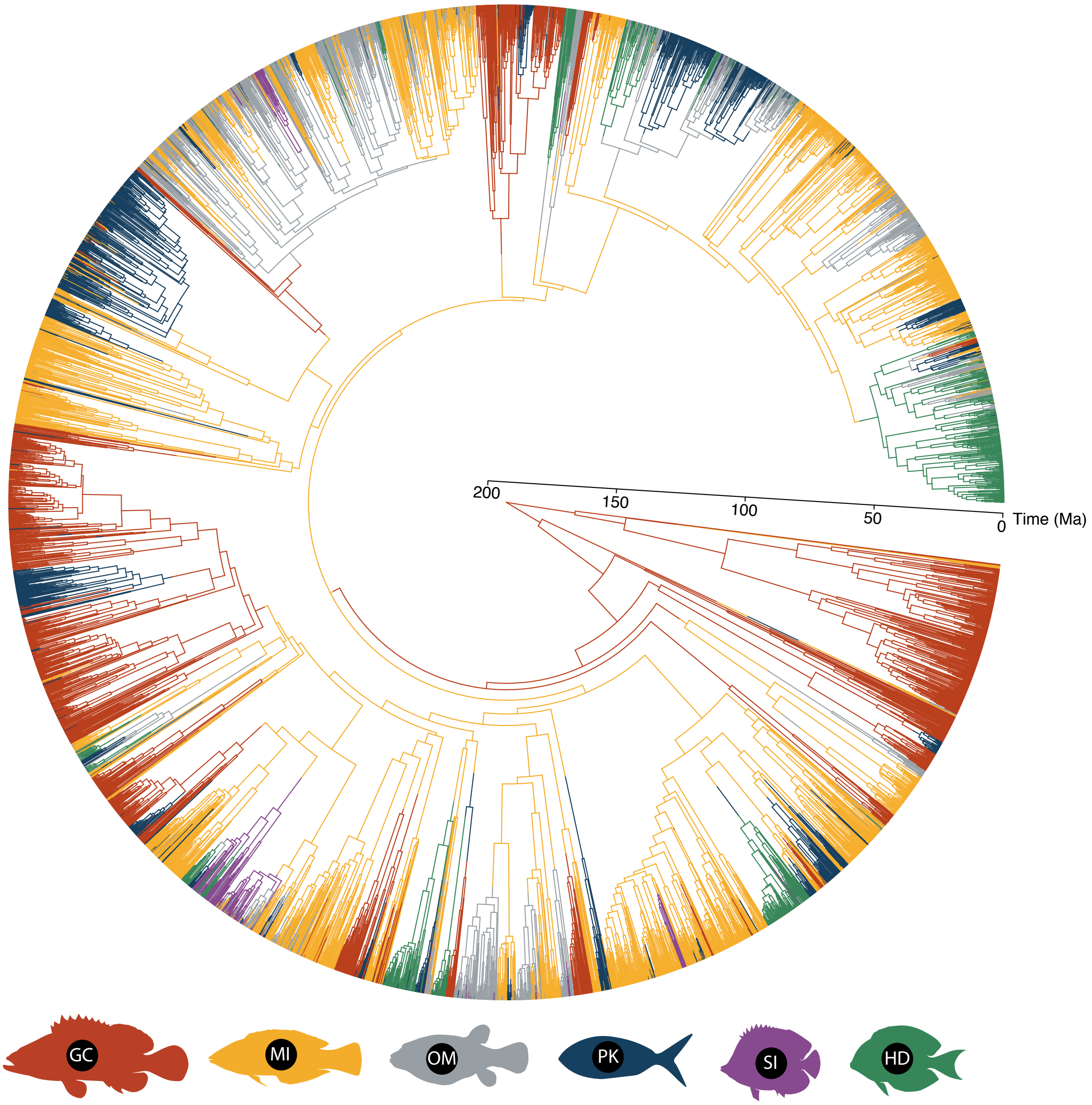

Supplementary Figure 4. Ancestral state reconstruction of reef fish trophic guilds. This reconstruction represents one of the stochastic character mappings. HD: herbivores/detritivores (green); SI: sessile invertivores (purple); OM: omnivores (grey); PK: planktivores (blue); MI: mobile invertivores (yellow); GC: generalized carnivores (red). Fish silhouettes were sourced from Nina M. D. Schiettekatte, Simon J. Brandl and Jordan M. Casey (2020). fishualize: Color Palettes Based on Fish Species. R package version 0.2.0. https://CRAN.R-project.org/package=fishualize.

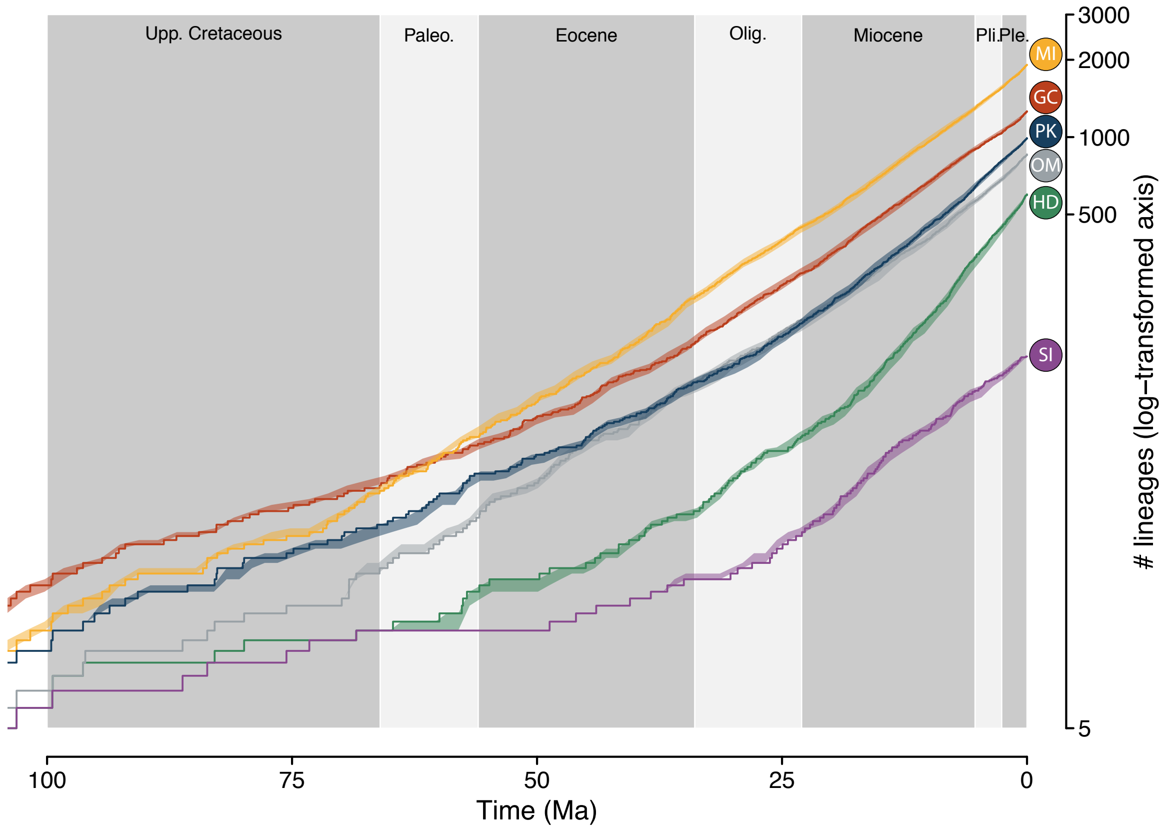

Supplementary Figure 5. Lineage through time plot for each reef fish trophic guild. Solid line derives from a randomly selected phylogenetic tree, while semi-transparent polygons represent the 95% confidence intervals derived from 100 trees. HD: herbivores/detritivores (green); SI: sessile invertivores (purple); OM: omnivores (grey); PK: planktivores (blue); MI: mobile invertivores (yellow); GC: generalized carnivores (red).

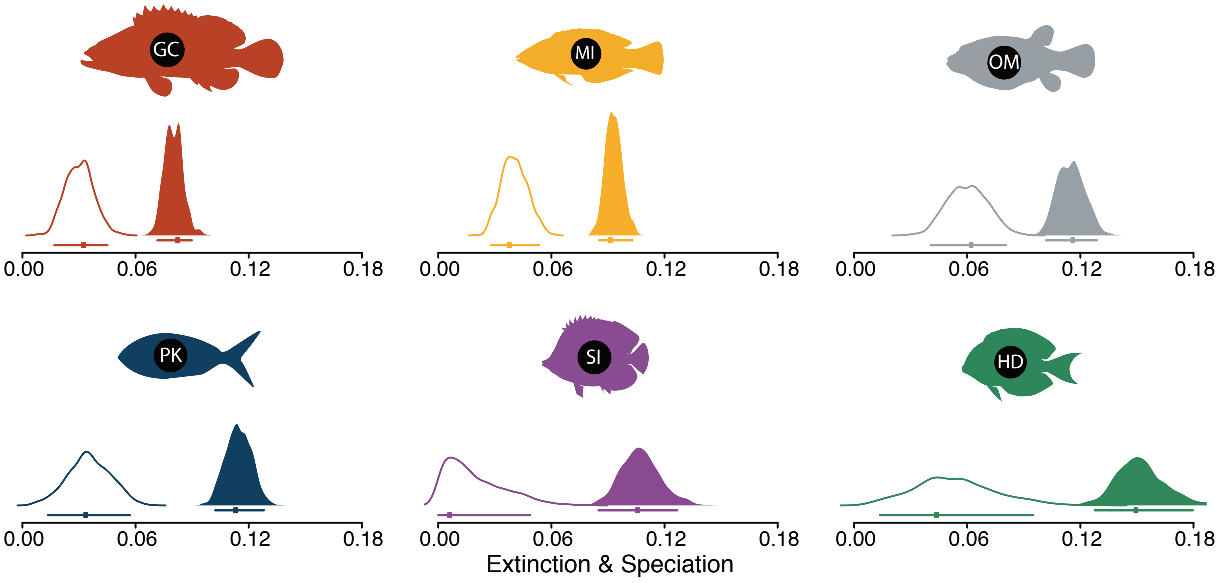

Supplementary Figure 6. Historical extinction and speciation rate estimates for each reef fish trophic group, derived from MuSSE. Lines below the distributions show mode values (solid circles) with respective 95% credibility intervals. HD: herbivores/detritivores (green); SI: sessile invertivores (purple); OM: omnivores (grey); PK: planktivores (blue); MI: mobile invertivores (yellow); GC: generalized carnivores (red). Fish silhouettes were sourced from Nina M. D. Schiettekatte, Simon J. Brandl and Jordan M. Casey (2020). fishualize: Color Palettes Based on Fish Species. R package version 0.2.0. https://CRAN.R-project.org/package=fishualize. Source data is provided as a Source Data file.

Supplementary tables

Supplementary Table 1. Model results derived from the HiSSE analysis ranked according to the lowest Akaike Information Criterion (AIC). The unconstrained model considered rates to be different between analysed character states (trophic group) with one hidden diversification regime per state, whereas the constrained model considers rates to be equal between analysed states but different from the hidden diversification regime. logLik: model likelihood; Net div ancs: net diversification for the ancestral trophic groups (generalized carnivores, mobile invertivores and omnivores); Net div rec: net diversification for the recently derived trophic groups (herbivores/detritivores, sessile invertivores and planktivores); Net div ancs H: hidden net diversification regime for the ancestral trophic groups; Net div rec H: hidden net diversification regime for the recently derived trophic groups.

	logLik	AIC	Net div ancs	Net div rec	Net div ancs H	Net div rec H
**Uncostrained**	-21736.8	43501.7	0.0253496	0.0945460	0.1764130	0.2314718
**Constrained**	-21859.7	43739.4	0.0426502	0.0426502	0.1432365	0.1432365
