# Supplementary material for: Trophic innovations fuel reef fish diversification
Source: Nat Commun. 2020 May 29;11:2669. doi: 10.1038/s41467-020-16498-w (PMC7260216; doi:10.1038/s41467-020-16498-w)
Supplement: Supplementary file 3 — Reporting Summary [file 41467_2020_16498_MOESM3_ESM.pdf]

nature research

Corresponding author(s): Alexandre C. Siqueira
Last updated by author(s): Apr 27, 2020

Reporting Summary

Nature Research wishes to improve the reproducibility of the work that we publish. This form provides structure for consistency and transparency in reporting. For further information on Nature Research policies, see Authors & Referees and the Editorial Policy Checklist .

Statistics

For all statistical analyses, confirm that the following items are present in the figure legend, table legend, main text, or Methods section.

n/a	Confirmed	
☐	☒	The exact sample size (*n*) for each experimental group/condition, given as a discrete number and unit of measurement
☐	☒	A statement on whether measurements were taken from distinct samples or whether the same sample was measured repeatedly
☐	☒	The statistical test(s) used AND whether they are one- or two-sided<br>*Only common tests should be described solely by name; describe more complex techniques in the Methods section.*
☐	☒	A description of all covariates tested
☐	☒	A description of any assumptions or corrections, such as tests of normality and adjustment for multiple comparisons
☐	☒	A full description of the statistical parameters including central tendency (e.g. means) or other basic estimates (e.g. regression coefficient) AND variation (e.g. standard deviation) or associated estimates of uncertainty (e.g. confidence intervals)
☐	☒	For null hypothesis testing, the test statistic (e.g. *F*, *t*, *r*) with confidence intervals, effect sizes, degrees of freedom and *P* value noted<br>*Give P values as exact values whenever suitable.*
☐	☒	For Bayesian analysis, information on the choice of priors and Markov chain Monte Carlo settings
☐	☒	For hierarchical and complex designs, identification of the appropriate level for tests and full reporting of outcomes
☐	☒	Estimates of effect sizes (e.g. Cohen's *d*, Pearson's *r*), indicating how they were calculated

Our web collection on statistics for biologists contains articles on many of the points above.

Software and code

Policy information about availability of computer code

Data collection	We used R scripts to extract and process data from multiple sources. The phylogenetic tree was downloaded from The Fish Tree of Life (https://fishtreeoflife.org); and most of the raw data was downloaded from the Dryad digital data repository (https://doi.org/10.5061/dryad.fc71cp4). Other publicly available datasets used in the study include: FishBase (http://www.fishbase.org/search.php), and Eschemeyer's Catalog of Fishes (http://researcharchive.calacademy.org/research/ichthyology/catalog/fishcatmain.asp). We used R version 3.5.3.
Data analysis	We used the softwares BAMM (version 2.5.0); TACT (version 5.2.4); and R (version 3.5.3) to perform data analyses. The R packages used were: ape (version 5.3); BAMMtools (version 2.1.6); coda (version 0.19.3); phytools (version 0.6.99); circlize (version 0.4.6); scales (version 1.0); diversitree (version 0.9.11); HDInterval (version 0.2.0); plyr (version 1.8.4); tidyverse (version 1.2.1); pdp (version 0.7.0); xgboost (version 0.82.1); hisse (version 1.9.6); rfishbase (version 3.0.4); fishualize (version 0.2.0).

For manuscripts utilizing custom algorithms or software that are central to the research but not yet described in published literature, software must be made available to editors/reviewers. We strongly encourage code deposition in a community repository (e.g. GitHub). See the Nature Research guidelines for submitting code & software for further information.

Data

Policy information about availability of data

All manuscripts must include a data availability statement. This statement should provide the following information, where applicable:

- Accession codes, unique identifiers, or web links for publicly available datasets
- A list of figures that have associated raw data
- A description of any restrictions on data availability

The datasets generated during and/or analysed during the current study are available at the James Cook University's Tropical Data Hub repository (https://doi.org/10.25903/5e9659dbca234). There are no restrictions on data availability. The phylogeny used as backbone was downloaded from The Fish Tree of Life (https://fishtreeoflife.org). Publicly available datasets used in the study include: FishBase (http://www.fishbase.org/search.php), Eschemeyer's Catalog of Fishes (http://researcharchive.calacademy.org/research/ichthyology/catalog/fishcatmain.asp), and the Dryad repository of Rabosky et al. (https://doi.org/10.5061/

dryad.fc71cp4). The source data underlying Figs 2a–b, 3a–d, 4, 5 and Supplementary Figs 1a–f, 2a–f, 3a–f and 6 are provided as a Source Data file.

Field-specific reporting

Please select the one below that is the best fit for your research. If you are not sure, read the appropriate sections before making your selection.

☐ Life sciences ☐ Behavioural & social sciences ☒ Ecological, evolutionary & environmental sciences

For a reference copy of the document with all sections, see nature.com/documents/nr-reporting-summary-flat.pdf

Ecological, evolutionary & environmental sciences study design

All studies must disclose on these points even when the disclosure is negative.

Study description	We tested ecological and geographical factors underlying the diversification rate patterns in reef fishes. Additionally, we analyzed the patterns of trophic evolution within this vertebrate group.
Research sample	The research sample was composed of fishes associated with reef habitats. We selected families with more than 20% of its species as reef-associated and collected ecological and geographic data for all of these species in publicly available datasets (see Data collection for sources).
Sampling strategy	Sample size was determined by availability of phylogenetic, ecological and geographic data for reef fishes. We used all available data in our study.
Data collection	The phylogenetic tree was downloaded from http://fishtreeoflife.org; and most of the raw data was downloaded from the Dryad digital data repository (https://doi.org/10.5061/dryad.fc71cp4). This repository refers to the paper of Rabosky et al. (2018; https://doi.org/10.1038/s41586-018-0273-1) and was publicly available. Additionally, we used data available in the publicly available FishBase and Eschemeyer's Catalog of Fishes datasets.
Timing and spatial scale	The scope of this study is global. Since all data used was publicly available, there were no time constraints for data collection.
Data exclusions	There were no data exclusions.
Reproducibility	Since our study is not an experiment, our results can not be replicated. However, we performed sensitivity analyses to make sure that our results were robust independently of method used.
Randomization	Organisms analyzed herein were chosen based on habitat and taxonomic affinities (Reef fishes).
Blinding	Blinding was not relevant to our study, because we did not perform an experiment.

Did the study involve field work? ☐ Yes ☒ No

Reporting for specific materials, systems and methods

We require information from authors about some types of materials, experimental systems and methods used in many studies. Here, indicate whether each material, system or method listed is relevant to your study. If you are not sure if a list item applies to your research, read the appropriate section before selecting a response.

Materials & experimental systems

n/a	Involved in the study	
☒	☐	Antibodies
☒	☐	Eukaryotic cell lines
☒	☐	Palaeontology
☒	☐	Animals and other organisms
☒	☐	Human research participants
☒	☐	Clinical data

Methods

n/a	Involved in the study	
☒	☐	ChIP-seq
☒	☐	Flow cytometry
☒	☐	MRI-based neuroimaging
