# Supplementary material for: Trophic innovations fuel reef fish diversification
Source: Nat Commun. 2020 May 29;11:2669. doi: 10.1038/s41467-020-16498-w (PMC7260216; doi:10.1038/s41467-020-16498-w)
Supplement: Supplementary file 2 — Peer Review [file 41467_2020_16498_MOESM2_ESM.pdf]

Reviewers' comments:

Reviewer #1 (Remarks to the Author):

The manuscript, “Trophic innovations fuel reef fish diversification” provides further insights into our understanding of the drivers of reef fish diversification and highlights the pivotal evolutionary role of trophic identity and body size. The manuscript is well written and will be of interest to any coral reef ecologists and evolutionary biologists. I only have few comments, which could be easily addressed by the authors. Here are some general comments:

1) Although the introduction is well structured and pleasant to read, there is one paragraph missing about the role of diet during the evolution of reef fishes, as it appears to be one of the main biotic factors explored in the present study (objectives at lines 70-76). This will help the reader to understand why it is important to focus on trophic guilds. Such a statement could be added at lines 54-56 where the authors mentioned that reef fishes are key energetic conduits.

2) The authors group several trophic guilds into the “generalized” category and others into the “specialized” category. Despite multiple references of generalized and specialized groups throughout the text (e.g., lines 163, line 165, line 180) and a brief description of which groups belong to which category in the abstract (lines 29-30), the authors gave no real explanation for this binary categorization. For example, it could be based on what types of food sources fishes are feeding on and/or from which compartment (water column versus benthos). Such a paragraph needs to be included in the introduction. Likewise, the authors mention trophic innovations several times in the main text and even in the title, but did not include any explanation of what constitutes a trophic innovation. Providing a definition or examples of novel feeding modes may help bringing some clarification.

3) There are also strong similarities between some results of the present study and those of Gajdzik et al. 2019. For example, here are some quotes from the present study: “…the role of omnivory as an intermediate evolutionary step between higher and lower trophic levels…” or “…leading the authors to flag omnivory as a macroevolutionary sink. Although this was not the case for reef fishes...” Here are some quotes from Gajdzik et al. 2019: “…the generalist carnivore guild in Labridae and the intermediate trophic guild in Pomacentridae may act as a stepping-stone state towards more specialised strategies” or “The generalist feeding guild is not an evolutionary sink”. Given the similarities between these two studies, it appears advisable to discuss about the results of Gajdzik et al. 2019 in the present study.

Overall, I recommend this manuscript for publication and would like to congratulate the authors.

 Other comments line by line:

Lines 53-54: Please provide some examples of the biotic interactions provided by coral reefs or be directly more specific.

Lines 89-92: There are two representations of the net diversification rate on Figure 1 (one in the center of the figure and a second one on the left). This is especially confusing considering that the values are different although they refer to the same measure (i.e., net diversification rate). Please either remove one representation or provide additional information for the reader to understand both of them.

Lines 95: Could the authors also provide the justification for the choice of the number of trophic guilds? Since trophic identity are not homogeneously distributed among fish families and that the evolutionary history of each fish family was influenced by other specific biotic parameters (e.g., trait decoupling; Frederich et al. 2014), it would be interesting to know whether the authors tested coarser

and finer trophic guild classifications and if so, to what extent it affected or not the outcomes of the manuscript. Additionally, trophic identity is not necessarily a fixed attribute and thus a short statement about how the authors dealt with species that shift diet in various locations should be provided if tested.

Lines 104: Please define the acronyms used in Figure 2 for the other ecological and geographical variables other than size and trophic traits. Please also explain the relevance for the other environmental data used in your extreme gradient boosting analysis since it is also part of your study (line 64). For example, salinity was not used in the model, but it varies greatly in some regions of the world (e.g., Red Sea; Dibattista et al. 2015). In addition, the choice of satellite products (e.g., NOAA, OI-SST) and its resolution (daily versus monthly) will affect the values of mean sea surface temperature and some issues have been recently raised (e.g., DeCarlo & Harrison 2019). Likewise, it would be interesting to know how the authors computed the centroid grid for range-shifting fish species – species expanding their geographic range that are more common in some families (e.g., Siganidae or Pomacentridae)- as it will impact the calculation of the mean sea surface temperature.

Line 192: Using chord diagram to illustrate trophic transitions is an excellent idea. However, it could be easier to understand the patterns of trophic transitions if there were only two circular plots (i.e., one for the specialized group and a second one for the generalized group) as well as one color per trophic identity. A legend is also needed in order to know which width corresponds to how many transitions.

Lines 210: It appears adequate to define what is a "trophic innovation" versus a "trophic specialization" or "ecological opportunity", especially that Lobato et al. 2014 did not use the term innovation but ecological opportunities (Please see my general comments above).

Lines 289-293: Alongside with citing the results of a mammal study and a bird study, it appears relevant to mention here the results of Gajdzik et al. 2019 on coral reef fishes, which discussed the role of generalized trophic strategies during the evolutionary of reef fish and found they were not evolutionary sinks (Please see my general comments above).

Lines 435-438: Please provide references of the literature, online datasets, and expert assessments used.

Lines 529-537: Although the authors acknowledge the issues with MuSSE, other tests could be done to alleviate them. One solution is to transform your data as binary (generalists versus specialists) and switch to a HiSSE test (Beaulieu & O'Meara 2016), which is by far the most robust method as it uses an appropriate multi-rate null but there is not an equivalent for multi-state data. Another way is to divide the dataset up into different clades as suggested by Rabosky & Goldberg et al. 2015. It appears interesting to explore one of these tests and see whether results are still similar to those of MuSSE.

Lines 540-544: Please provide the parametrisation of the stochastic mapping.

References

Beaulieu, J.M., & O'Meara, B.C. (2016). Detecting hidden diversification shifts in models of trait-dependent speciation and extinction. Systematic Biology, 65(4): 583–601.

DiBattista, J.D., Roberts, M.B., Bouwmeester, J., Bowen, B.W., Coker, D.J., Lozano-Cortés, D.F., Howard Choat, J., Gaither, M.R., Hobbs, J.-P.A., Khalil, M.T., Kochzius, M., Myers, R.F., Paulay, G., Robitzch, V.S.N., Saenz-Agudelo, P., Salas, E., Sinclair-Taylor, T.H., Toonen, R.J., Westneat, M.W., Williams, S.T. & Berumen, M.L. (2016). A review of contemporary patterns of endemism for shallow water reef fauna in the Red Sea. Journal of Biogeography, 43: 423-439.

DeCarlo, TM, & Harrison, HB. (2019) An enigmatic decoupling between heat stress and coral bleaching on the Great Barrier Reef. PeerJ 7: e7473

Frederich, B., Damien, O., Litsios, G., Alfaro, M.E., & Parmentier, E. (2014). Trait decoupling promotes evolutionary diversification of the trophic and acoustic system of damselfishes. Proc. R. Soc. B. 281.

Gajdzik, L., Aguilar-Medrano, R. & Frédérich, B. (2019). Diversification and functional evolution of reef fish feeding guilds. Ecology Letters, 22: 572-582.

Rabosky D.L. & Golberg E.G. (2015). Model inadequacy and mistaken inferences of trait-dependent speciation. Systematic Biology, 64 (2): 340–355.

Reviewer #2 (Remarks to the Author):

Siqueira and colleagues present a new study of the drivers of diversification in reef fishes, and find a link to trophic innovations, specifically herbivory, during a Miocene event. While this is an interesting result and the authors have undertaken extensive efforts to test their hypotheses and their results have strong support, their starting assumptions, methodological choices and favored and alternative interpretations do not seem to have received as much scrutiny. Their points need a lot more justification and explanation.
To start, the study claims to focus on reef fishes, yet their trees include all members of families which contain at least 20% reef-associated taxa. This potentially drowns out signal in favor of sample size. As another example, they note that inferring extinction rates from trees containing only living taxa is problematic, particularly for small fishes, but then report high extinction rates for Omnivores which they also find are not evolutionarily stable. In other words, potential confounders are reported but not fully taken on board. Finally, they report that their diversification pulses are likely associated with a Miocene event, such as rearrangement of reef systems, but do not report the timing or test it. Further, their tree may not be temporarily resolved enough to detect clustered divergences from 8 million years ago, given that it was pruned from a much larger tree that used fossil calibrations from a much longer time period. Recalibration using fossils from the genera and families themselves is necessary given the more recent focus. Finally, there is not much explanation of their results in terms of ecology and diversification triggers. They find that herbivores are generally larger and show high diversification rates, but do not explain what the mechanism might be beyond generalized predation pressure, why this might be given their large ranges, or how this lines up with the Miocene event they propose.
That said, I think this study is a worthy undertaking, the aims are well planned, and the results could be really informative and interesting. The issues above and in specific comments before they can really be taken on board.

Specific Comments below:

Larger size may not just be about predation pressure, aren’t herbivores generally larger? How is the plant matter processed?

What about growth rates? Herbivorous reef fishes can take years to reach adult size, and are fulfilling all herbivorous size-niches up to that point. Maybe it’s more about making up for losses (e.g. Ocean sunfish)? Larger individuals produce more eggs, enough to make up for losses?

Why would herbivores have high diversification rates if they also have large ranges?

The link with a Miocene diversification event seems weak – is there at least a way to illustrate the timing of diversification in line with this event? In addition, it is not made clear in this manuscript why reef rearrangement in Miocene happened or why this should influence fish diversification. The Miocene is also the time of onset of polar glaciation and increased latitudinal diversity gradients, and may be associated with a decline in diversification in the tropics (Rabosky et al., 2018). Thus, it seems a less than obvious time for reef fish diversification to increase.

-288: Why are extinction rates slightly higher for omnivores? Are they really extinction rates if omnivory is transitional between other states, or is just that such lineages don't exist in that space for long? Line 308 says estimating extinction rates without fossils is problematic for small fishes and gobies but MuSSE was the only analysis applied regardless. Line 341 points out omnivory is not evolutionarily stable.

359: Could other factors be sociality and sexual selection, given that the herbivorous fishes tend to live in large groups?

363: Evidence for herbivores triggering Miocene reef formation seems circumstantial. This needs more information on patterns of reef building. What about Eocene reef-building? Early herbivores are found at places like Monte Bolca, where reef fishes exhibit more disparity than modern fishes (see papers). Couldn't this result come from the use of living fishes alone, representing a turnover event rather than initial reef-building?

Methods:

Reef fish phylogeny: Wouldn't it have been better to use data from FishBase to prune the tree to contain only specific reef-associated taxa rather than entire families that are at least 20% reef-associated (which seems really low when you are test reef-associated diversification, it seems like most coastal fishes would end up included)? It seems like this approach is confounded by diversification patterns shaped by the conditions of alternative habitats, or the influence of transients. This would have produced a smaller tree but possibly stronger results.

It also seems like the tree should have been recalibrated at a lower level, since the original tree contained 139 fossils for all ray-fins. The first occurrences of specific genera would have allowed for better estimation of diversification rates considering the event of interest (Miocene reef building) is only 8 million years out. How do you know your divergence times are realistic for this subset?

470: The methods in section d need more explanation in text for why this machine learning approach is preferred over prior or other existing modelling approaches, rather than a reference to a different paper followed by parameter selection criteria.

505: What is a "consensus family"?

516: Extinction estimated using MuSSE. What about the issues with estimating extinction in trees containing only living taxa? Same with diversification rates.

540: Which function was used in Phytools for stochastic character mapping?

Reviewer #3 (Remarks to the Author):

This paper aims to explore the relative importance of ecological and geographical factors explaining heterogeneity in the tempo of lineage diversification among major reef-associated fish families. Along this work, the authors applied cutting-edge phylogenetic comparative methods on near complete phylogenetic trees retrieved from Rabosky et al. (2018).

At the first glance, it is rather intuitive that feeding habits would be a major driver of diversification in the hyper diverse ichthyofauna living in reef ecosystems. So, I am not really surprised by the main results of the present study. However, even if many ichthyologists likely share my opinion, the authors made a good job by providing strong supports on this hypothesis at a very large taxonomic fish scale. Section (b) of results (lines 94-158) are probably the most satisfying ones of the present MS but I am much more concerned about the section (c) of results (lines 160-197). Accordingly, I have listed some comments that should be addressed prior to an acceptance for publication.

Specific Comments:

Introduction:
The introductory part is broadly oriented, mainly suggesting that various biotic and abiotic factors could sustain variation in the tempo of reef fish lineage diversification. To my opinion, the authors should work better to introduce (their) expectations about the impact of feeding novelties and size variation on lineage diversification. Various studies already showed that trophic attributes acted as a major driving force during reef-associated fish evolution (e.g. Price et al. 2010, Frédérich et al. 2013, Friedman et al. 2016, Borstein et al. 2019). I encourage the authors to use these studies to build some assumptions about the link between trophic evolution and diversification (taxonomic, morphological and functional diversity) in reef fishes.

Results and Discussion:
Results related to figures 1, 2 & 3 are the strength of the present paper. On the other hand, the authors failed to connect their results from figures 4 & 5 to a recent paper published in Ecology Letters by Gajdzik et al. (2019). Even if Gajdzik and colleagues only studied Labridae and Pomacentridae (in comparison to the larger sampling of fish families of the present study), they already studied variation in the tempo of lineage diversification among trophic guilds. They also illustrated the pivotal role of a generalist feeding strategy during the evolutionary history of reef-associated fishes.

This « oversight » is detrimental. Indeed, when the authors will include the work of Gajdzik et al. (2019) in their list of references and their considerations, 40% of the results from the present MS will not appear as « new » as expected. The miss of such a key paper does not fit the standard of bibliographic works required for scientific research in general and the expectations from a journal like Nature Communications. I urge the authors to include the work of Gajdzik et al. (2019) in their paper and re-work their introduction, results and discussion accordingly.

Page 4, line 81: Define « net diversification rate » (= speciation - extinction) for its first reference in the text.

Page 7, lines 125-126: How do the authors identify three size categories? This appears really unclear to me.

Page 7, line 129: The authors should be cautious when using terms like « specialization », « specialized »,… How a group made of detritivorous, macroalgivorous and filamentous algivorous species would be more specialized than groups made of omnivorous or carnivorous species?? Definition of specialization can be tricky and the authors' definition of specialization have to be clearly stated.

Page 12, lines 200-202: Many other references could be added here. Please, avoid self-citations and

add, for example, some of the references cited above (and other papers).

Page 12, lines 210-215: See results from Gajdzik et al. (2019) and include them in your discussion.

Page 13, lines 245-256: Please, provide more explanations on why the expansion of corals in shallow waters may sustain the diversification of sessile invertivores and planktivores. How does water flow vary during/after the rise of reef flat?

Page 14, lines 257-282: Discussion about natural selection on large body sized herbivores is interesting but highly speculative. The authors should shorten this section of the discussion but they should suggest some tips for testing this in next works. As an example, models of size evolution (BM - Brownian motion and multiple OU - Ornstein Uhlenbeck models) could be fitted and compared in order to assess an optimum for large sized grazing fishes on reef flat habitat.

Pages 15-17: All this part of the discussion should be re-worked by including the results from Gajdzik et al. (2019).

Page 17, lines 323-326: This sentence is unclear. What does « departures from typical morphologies and behaviors towards planktivory » mean?

A great part of the results/discussion focus on the diversification of large herbivorous/detritivores fishes. However BAMM analyses also revealed high rates of lineage diversification in snappers (Lutjanidae) and butterflyfishes (Chaetodontidae). Then, this information is diluted in the rest of the text. How the high rates of diversification in both families are supported by MuSSE results? What can explain such high rates of net diversification in both families which include fish species feeding on animal prey?

Figures: why do the authors chose silhouette from different fish families for illustrating trophic strategies? It is not really appropriate. It is misleading because, first, we could primarily associated silhouettes to taxonomic groups (fish family). Moreover we know that one fish family generally includes more than one trophic strategy. So, please, modify this iconography.

Cited references

Borstein, S.R., Fordyce, J.A., O'Meara, B.C., Wainwright, P.C., and McGee, M.D. (2019). Reef fish functional traits evolve fastest at trophic extremes. Nature Ecology and Evolution 3, 191-199

Gajdzik, L., Aguilar-Medrano, R., and Frédérich, B. (2019). Diversification and functional evolution of reef fish feeding guilds. Ecology Letters 22, 572-582.

Frédérich, B., Sorenson, L., Santini, F., Slater, G.J., and Alfaro, M.E. (2013). Iterative Ecological Radiation and Convergence during the Evolutionary History of Damselfishes (Pomacentridae). American Naturalist 181, 94-113.

Friedman, S.T., Price, S.A., Hoey, A.S., and Wainwright, P.C. (2016). Ecomorphological convergence in planktivorous surgeonfishes. Journal of Evolutionary Biology 29, 965-978.

Price, S.A., Wainwright, P.C., Bellwood, D.R., Kazancioglu, E., Collar, D.C., and Near, T.J. (2010). Functional innovations and morphological diversification in parrotfish. Evolution 64, 3057-3068.

Rabosky, D.L., Chang, J., Title, P.O., Cowman, P.F., Sallan, L., Friedman, M., Kaschner, K., Garilao, C., Near, T.J., Coll, M., et al. (2018). An inverse latitudinal gradient in speciation rate for marine fishes. Nature 559, 392-395.

Authors' replies to the comments made by Reviewer #1

Comment#1 (R#1): The manuscript, "Trophic innovations fuel reef fish diversification" provides further insights into our understanding of the drivers of reef fish diversification and highlights the pivotal evolutionary role of trophic identity and body size. The manuscript is well written and will be of interest to any coral reef ecologists and evolutionary biologists. I only have few comments, which could be easily addressed by the authors.

Author's reply: Thank you for your time and attention with our manuscript, we really appreciate your positive feedback. We have carefully reviewed and addressed all your suggestions. Below you will find more detailed responses explaining how we dealt with each comment. Note that line numbers refer to the revised version of the manuscript with inline track changes.

Comment#2 (R#1): Although the introduction is well structured and pleasant to read, there is one paragraph missing about the role of diet during the evolution of reef fishes, as it appears to be one of the main biotic factors explored in the present study (objectives at lines 70-76). This will help the reader to understand why it is important to focus on trophic guilds. Such a statement could be added at lines 54-56 where the authors mentioned that reef fishes are key energetic conduits.

Author's reply: As suggested, we have now added a new paragraph in our introduction highlighting the potential role of trophic innovations in influencing reef fish diversification during the Miocene (Lines 69-82). This paragraph aids the understanding of our objectives and also introduces the ideas that are developed throughout the discussion.

Comment#3 (R#1): The authors group several trophic guilds into the "generalized" category and others into the "specialized" category. Despite multiple references of generalized and specialized groups throughout the text (e.g., lines 163, line 165, line 180) and a brief description of which groups belong to which category in the abstract (lines 29-30), the authors gave no real explanation for this binary categorization. For example, it could be based on what types of food sources fishes are feeding on and/or from which compartment (water column versus benthos). Such a paragraph needs to be included in the introduction. Likewise, the authors mention trophic innovations several

times in the main text and even in the title, but did not include any explanation of what constitutes a trophic innovation. Providing a definition or examples of novel feeding modes may help bringing some clarification.

Author's reply: We agree that the terms 'generalized' and 'specialized' would require clearer a definition, which was lacking in the first version of our manuscript. This is a very contentious topic, and to avoid confusion we have removed all the references to 'generalized' and 'specialized' throughout the text. In some cases, we now make a reference to the fact that the previously termed 'specialized' trophic groups are evolutionarily more recent, which is a definition that captures the ideas we were originally trying to explain. Additionally, we now provide an explicit definition of trophic innovations in the new paragraph of the introduction (Lines 75-76). This clarifies the use of the term in the title and throughout the text. Thank you for helping us to clarify these issues.

Comment#4 (R#1): There are also strong similarities between some results of the present study and those of Gajdzik et al. 2019. For example, here are some quotes from the present study: "…the role of omnivory as an intermediate evolutionary step between higher and lower trophic levels…" or "…leading the authors to flag omnivory as a macroevolutionary sink. Although this was not the case for reef fishes..." Here are some quotes from Gajdzik et al. 2019: "…the generalist carnivore guild in Labridae and the intermediate trophic guild in Pomacentridae may act as a stepping-stone state towards more specialised strategies" or "The generalist feeding guild is not an evolutionary sink". Given the similarities between these two studies, it appears advisable to discuss about the results of Gajdzik et al. 2019 in the present study.

Author's reply: Thank you for bringing this important reference to our attention. By focusing on the large-scale results, we might have missed the details of previous studies that targeted selected reef fish families. Indeed, some of the transition results found for Pomacentridae and Labridae in Gajdzik *et al.*, (2019) are complementary to our results for the full reef fish tree. We have now cited this reference wherever we felt a citation was relevant to highlight related findings (Lines 84-86; 230-231; 337-340; 382-384; 387-391).

Comment#5 (R#1): Overall, I recommend this manuscript for publication and would like to congratulate the authors.

Author's reply: Thank you for your overall evaluation and constructive comments.

Comment#6 (R#1): Lines 53-54: Please provide some examples of the biotic interactions provided by coral reefs or be directly more specific.

Author's reply: We agree that the highlighted sentence was vague. To avoid confusion, we have removed this part of the sentence (Lines 55-57).

Comment#7 (R#1): Lines 89-92: There are two representations of the net diversification rate on Figure 1 (one in the center of the figure and a second one on the left). This is especially confusing considering that the values are different although they refer to the same measure (i.e., net diversification rate). Please either remove one representation or provide additional information for the reader to understand both of them.

Author's reply: We agree with the reviewer that two representations of diversification rate in Figure 1 were unnecessary for interpreting the figure. We have, therefore, simplified the figure and removed the representation on the top left corner.

Comment#8 (R#1): Lines 95: Could the authors also provide the justification for the choice of the number of trophic guilds? Since trophic identity are not homogeneously distributed among fish families and that the evolutionary history of each fish family was influenced by other specific biotic parameters (e.g., trait decoupling; Frederich et al. 2014), it would be interesting to know whether the authors tested coarser and finer trophic guild classifications and if so, to what extent it affected or not the outcomes of the manuscript. Additionally, trophic identity is not necessarily a fixed attribute and thus a short statement about how the authors dealt with species that shift diet in various locations should be provided if tested.

Author's reply: This is an interesting comment. The number of trophic guilds analyzed in our study was based on commonly used categories to capture the main types of diets in reef fish adult life stages (e.g. Mouillot *et al.*, 2014; Parravicini *et al.*, 2014). The main difference in our scheme is that we merged the herbivores/macroalgivores category with the general herbivores/detritivores group, given the small sample size of the first, to avoid an extremely

unbalanced design that could result in biases in the models. These details on the dietary categories are provided in our methods section (Lines 489-502). Although this could be considered a coarse classification of diets, it is particularly useful for large scale analysis because it allows comparisons between disparate evolutionary lineages. We agree that finer classification schemes are possible, however, they would not provide enough resolution to test our hypotheses. Finer schemes could be used in future family-level studies to bring a more nuanced understanding of particular trophic innovations. Likewise, geographical diet shifts have not been considered in our study given that it has only been reported in a relatively small proportion of species analysed. Our classification considered the most common diets described for each species, which, given the broad categories, encompass most of the potential geographical variation. We have now added a sentence in the Methods section (Lines 501-502) clarifying this point.

Comment#9 (R#1): Lines 104: Please define the acronyms used in Figure 2 for the other ecological and geographical variables other than size and trophic traits. Please also explain the relevance for the other environmental data used in your extreme gradient boosting analysis since it is also part of your study (line 64). For example, salinity was not used in the model, but it varies greatly in some regions of the world (e.g., Red Sea; Dibattista et al. 2015). In addition, the choice of satellite products (e.g., NOAA, OI-SST) and its resolution (daily versus monthly) will affect the values of mean sea surface temperature and some issues have been recently raised (e.g., DeCarlo & Harrison 2019). Likewise, it would be interesting to know how the authors computed the centroid grid for range-shifting fish species – species expanding their geographic range that are more common in some families (e.g., Siganidae or Pomacentridae)- as it will impact the calculation of the mean sea surface temperature.

Author's reply: Thank you for noting the absence of acronym definitions, these have now been added to the legend of Figure 2 (Lines 131-133). Additionally, we now explain the relevance of the environmental data used in our gradient boosting analysis (Lines 517-519; 523-525). The choice of these variables was based on previous hypotheses about the influence of biogeography (e.g. Cowman & Bellwood, 2011; Bellwood *et al.*, 2017) or factors related to latitude (e.g. Siqueira *et al.*, 2016) on reef fish diversification rates. Although salinity does vary across marine biogeographical regions, this variability mainly concerns relatively

isolated systems that have suffered multiple extinction events and, thus, have likely a small contribution to large-scale historical rates of diversification (Dibattista *et al.*, 2016). Furthermore, to our knowledge, there are no explicit hypotheses linking it to the rates of evolution in reef fishes.

Regarding satellite products, we agree that the choice might influence values of mean sea surface temperature, particularly in local spatial scales as shown by DeCarlo & Harrison, (2019). However, in global analyses relying on data collected across large geographical and temporal scales (such as Rabosky *et al.*, 2018 and the present study), local measurement imprecisions are unlikely to influence results if potential biases are relatively constant across the regions evaluated. Although some satellite products are likely to show differential latitudinal bias (e.g. Bio-ORACLE products, Tyberghein *et al.*, 2012), these occur only when data from extreme latitudes (60-90 degrees) is included, which is not the case of our study which is restricted to low latitudes.

In the case of range-shifting species, we appreciate that some range extensions could slightly alter the calculated centroid of the species distribution. However, it is unlikely that this would influence our results because these species comprise a very small proportion of the species analysed in our study. Furthermore, range shifts are an emerging phenomenon only now starting to be quantified compared to historical range shifts which have been compiled throughout decades (e.g. IUCN red list distribution maps made by recognised experts in the field). Indeed, the hypothesis implied on your comment, i.e. that range-shifting species may change the future diversification patterns of their lineages, is an interesting one although impossible to test at this point.

Comment#10 (R#1): Line 192: Using chord diagram to illustrate trophic transitions is an excellent idea. However, it could be easier to understand the patterns of trophic transitions if there were only two circular plots (i.e., one for the specialized group and a second one for the generalized group) as well as one color per trophic identity. A legend is also needed in order to know which width corresponds to how many transitions.

Author's reply: Thank you for commending our Figure 5. We do appreciate the idea of keeping the figure simple by making only two circular plots. However, since we are now avoiding the binary classification between 'specialised' and 'generalised' groups (comment

#3), we decided to keep the figure depicting trophic groups separately. This also helps visualizing the patterns described in our results. However, to comply with your other suggestions, we kept only one colour per trophic group and added a scale to the width of the transitions.

Comment#11 (R#1): Lines 210: It appears adequate to define what is a "trophic innovation" versus a "trophic specialization" or "ecological opportunity", especially that Lobato et al. 2014 did not use the term innovation but ecological opportunities (Please see my general comments above).

Author's reply: We now provide an explicit definition for the term trophic innovation in our restructured introduction (Lines 75-76). Our concept follows the definition of 'evolutionary breakthroughs that change the adaptive landscape' reviewed by Wainwright (2007), being applied in a trophic context. This concept differs from 'ecological opportunity' used in Lobato *et al.*, (2014) because it focuses on the evolution of organismal traits rather than focusing on the environmental features of the system in which organisms are evolving. The definition of 'trophic specialization' is no longer necessary, since we removed those terms as noted above (see comment #3).

Comment#12 (R#1): Lines 289-293: Alongside with citing the results of a mammal study and a bird study, it appears relevant to mention here the results of Gajdzik et al. 2019 on coral reef fishes, which discussed the role of generalized trophic strategies during the evolutionary of reef fish and found they were not evolutionary sinks (Please see my general comments above).

Author's reply: Once again, thank you for suggesting this reference. It has now been included in the suggested part of the discussion (Lines 337-340) and in other sections of the manuscript.

Comment#13 (R#1): Lines 435-438: Please provide references of the literature, online datasets, and expert assessments used.

Author's reply: We now provide relevant references for our classification scheme (Line 490).

Comment#14 (R#1): Lines 529-537: Although the authors acknowledge the issues with MuSSE, other tests could be done to alleviate them. One solution is to transform your data as binary (generalists versus specialists) and switch to a HiSSE test (Beaulieu & O'Meara 2016), which is by far the most robust method as it uses an appropriate multi-rate null but there is not an equivalent for multi-state data. Another way is to divide the dataset up into different clades as suggested by Rabosky & Goldberg et al. 2015. It appears interesting to explore one of these tests and see whether results are still similar to those of MuSSE.

Author's reply: Thank you for this suggestion. We have performed the HiSSE analysis to alleviate the issues with MuSSE by splitting trophic groups between "ancestral" and "recently derived" as suggested. We highlight that the HiSSE results agree with the ones from MuSSE in the sense that diversification rates are higher in "recently derived" trophic groups. These results have been incorporated as a supplementary table in the new version of the manuscript (supplementary table S1) and the details of the analysis have been added to the methods section (Lines 602-612).

Comment#15 (R#1): Lines 540-544: Please provide the parametrisation of the stochastic mapping.

Author's reply: The stochastic mappings were performed with an ARD transition model and an empirical transition rate matrix. We have now added these parametrization details in the suggested methods section (Lines 615-618).

Authors' replies to the comments made by Reviewer #2

Comment#1 (R#2): Siqueira and colleagues present a new study of the drivers of diversification in reef fishes, and find a link to trophic innovations, specifically herbivory, during a Miocene event. While this is an interesting result and the authors have undertaken extensive efforts to test their hypotheses and their results have strong support, their starting assumptions, methodological choices and favored and alternative interpretations do not seem to have received as much scrutiny. Their points need a lot more justification and explanation.

Author's reply: Thank you for your feedback, we appreciate the attention given to our work. We carefully analysed your suggestions and modified the manuscript accordingly. Our approach to addressing each comment is detailed in our responses below. Please note that line numbers refer to the revised version of the manuscript with inline track changes. This revised version has considerably expanded the details on hypothesis, concepts and methods used and our hope that you find it suitable for publication.

Comment#2 (R#2): To start, the study claims to focus on reef fishes, yet their trees include all members of families which contain at least 20% reef-associated taxa. This potentially drowns out signal in favor of sample size.

Author's reply: Our goal by including all members of families containing at least 20% of reef-associated taxa was to have a tree that would be as comprehensive as possible. Although we agree that this means having non-reef taxa included in our study, this approach was very important to provide realistic estimates of diversification rates. As recently demonstrated by Chang *et al.*, (2019), incompletely sampled phylogenies can lead to biased estimates of diversification rates. Therefore, in our study we followed the recommendations of Chang *et al.* (2019) and use their algorithm (Taxonomic Addition for Complete Trees [TACT]) to improve our power in estimating rates of diversification. In addition, we performed an analysis considering only the "consensus" reef fish families (i.e. taxa that universally occur on coral reefs, rather than 'reefs' *sensu lato*; see comment #18). This analysis revealed a slight increase in model precision (36%) with trophic identity increasing in importance (56%) when compared to other variables (results section Lines 176-181; supplementary fig. 2). As noted in our discussion, having this as a supplementary analysis was important to show that our diversification rate results were most strongly associated with the history of coral reefs and not with peripheral environments that also support 'reef-associated' fish species (Lines 406-408).

Comment#3 (R#2): As another example, they note that inferring extinction rates from trees containing only living taxa is problematic, particularly for small fishes, but then report high

extinction rates for Omnivores which they also find are not evolutionarily stable. In other words, potential confounders are reported but not fully taken on board.

Author's reply: We agree that these two sentences of the discussion might appear to contradict each other. We have, therefore, restructured our paragraph that discusses the Omnivores (Lines 327-340) to eliminate this potential confusion. Please, see responses to comments #12 and #19 for more details on this issue.

Comment#4 (R#2): Finally, they report that their diversification pulses are likely associated with a Miocene event, such as rearrangement of reef systems, but do not report the timing or test it.

Author's reply: We now provide a figure to support the text that explicitly depicts the timing of lineage diversification per trophic groups (supplementary fig. S5). This lineage through time plot shows that reef fish diversification peaked in the Miocene, particularly in herbivores/detritivores, planktivores and sessile invertivores. Please, see response to comment #11 for more details related to this comment.

Comment#5 (R#2): Further, their tree may not be temporarily resolved enough to detect clustered divergences from 8 million years ago, given that it was pruned from a much larger tree that used fossil calibrations from a much longer time period. Recalibration using fossils from the genera and families themselves is necessary given the more recent focus.

Author's reply: We understand your point, however, even though we pruned our tree from the larger phylogeny of Rabosky *et al.* (2018), their calibration scheme used fossils from multiple time strata and their divergence dates match those from most previous studies at the genus level for multiple reef fish families. Please, see comment #16 for more details on this issue.

Comment#6 (R#2): Finally, there is not much explanation of their results in terms of ecology and diversification triggers. They find that herbivores are generally larger and show high diversification rates, but do not explain what the mechanism might be beyond generalized

predation pressure, why this might be given their large ranges, or how this lines up with the Miocene event they propose.

Author's reply: Firstly, it is important to clarify that we do not claim that herbivores are generally larger in our manuscript. What our results suggest is that large bodied herbivorous fishes have higher diversification rates when compared to small-medium sized herbivores and when compared to other trophic groups (Fig. 3A). Although we do propose a mechanistic explanation for higher diversification rates in large sized herbivores linked with predation pressure, we also discuss other mechanisms related to long-distance colonization capabilities (Lines 315-326). Larger body sizes have been shown to correlate positively with geographical range in reef fishes (Luiz *et al.*, 2013). This increases the chances of vicariance in large bodied fishes, given the variability in effectiveness of marine biogeographical barriers through time and the likelihood of fragmentation of previously contiguous populations (Lines 317-320). All of these processes align with the Miocene event proposed because once herbivorous fishes invaded reef flats, they are likely to have expanded their population sizes given the high productivity of this reef zone (Lines 281-282). This expansion might have been even more pronounced in large-bodied species given the predation process explained (Lines 298-314). Finally, the higher long-distance colonization capabilities of large bodied herbivorous fishes might have resulted in more vicariant events through time. It is our hope that we have now clarified this sequence of events throughout our discussion.

Comment#7 (R#2): That said, I think this study is a worthy undertaking, the aims are well planned, and the results could be really informative and interesting. The issues above and in specific comments before they can really be taken on board.

Author's reply: Thank you for highlighting the value of this study. We sincerely appreciate your encouragement.

Comment#8 (R#2): Larger size may not just be about predation pressure, aren't herbivores generally larger? How is the plant matter processed?

Author's reply: We agree that larger sizes might not just be related to predation pressure, therefore, we now reduced this part of the discussion (Lines 298-314) and also suggest a

potential role for long-distance colonization capabilities (Lines 315-326; comment #10). However, we would like to clarify that herbivores are not generally larger than other trophic groups in marine systems (see auxiliary figure below).

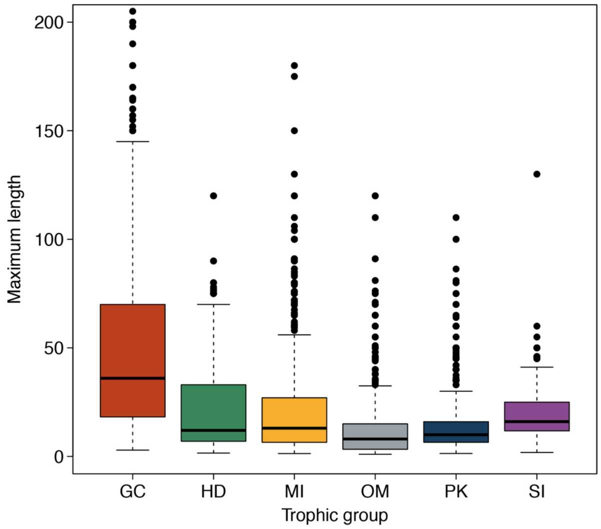

Auxiliary figure. Maximum body length distribution between reef fish species per classified trophic groups. Boxes represent upper and lower quartiles, and black lines show the median values. HD: herbivores/detritivores (green); SI: Sessile invertivores (purple); OM: omnivores (grey); PK: planktivores (blue); MI: mobile invertivores (yellow); GC: generalized carnivores (red).

Plant matter is processed differently in different herbivorous taxonomic groups. Using the most common herbivorous fish groups on coral reefs as examples, surgeonfishes (Acanthuridae) and rabbitfishes (Siganidae) mainly rely on stomach features for initial food processing. Their stomachs are usually divided between thin-walled or gizzard-like. Thin-walled stomachs are associated with acid lysis of food items and are found in rabbitfishes and some surgeofishes (*Naso* and some *Acanthurus*), while gizzard-like stomachs rely on thick, muscly walls to triturate food and are found in detritivorous surgeonfishes (*Ctenochaetus* and some *Acanthurus*). Some surgeonfishes and rabbitfishes also break food particles through fermentation within the hindgut. In parrotfishes (Scarini), on the other hand, stomachs are absent and they rely on a specialized pharyngeal jaw structure, known as pharyngeal mill, for triturating food particles. Finally, while parrotfishes absorb nutrients through sacculate intestines, surgeonfishes and rabbitfishes rely on very long intestines for nutrient absorption. For more details on plant matter processing by herbivorous fishes, please see Horn (1989). Some of these specialized morphologies for food processing (e.g.

gizzard-like stomachs) are encompassed within our broad term "trophic innovations", therefore, we have now included a sentence in the introduction that clarifies its use (Lines 74-82).

Comment#9 by (R#2): What about growth rates? Herbivorous reef fishes can take years to reach adult size, and are fulfilling all herbivorous size-niches up to that point. Maybe it's more about making up for losses (e.g. Ocean sunfish)? Larger individuals produce more eggs, enough to make up for losses?

Author's reply: Growth rates and reproductive outputs are indeed interesting aspects of reef fish biology. However, there is no clear link to higher diversification rates of large bodied herbivores. This is because larger individuals of fish produce more eggs, irrespective of trophic identity (Barneche *et al.*, 2018). Therefore, if higher egg production in larger species was related to higher diversification rates, we would have found enhanced diversification in large bodied species in all trophic groups and not only in herbivorous fishes. If growth rates are related to our results, it might be by reinforcing the predation process suggested in the discussion (Lines 287-302). All else being equal, herbivorous fishes tend to grow faster than other trophic groups (Morais & Bellwood, 2018), sometimes reaching the adult sizes in less than two years (e.g. Choat & Axe, 1996). This suggests that reaching a size refuge from predation could be a priority in the early life stages of some herbivorous fishes. Thank you for this interesting, thought provoking, question.

Comment#10 (R#2): Why would herbivores have high diversification rates if they also have large ranges?

Author's reply: This process is explained in more detail on the discussion (Lines 315-326). The key to understanding it lies in the fact that, in evolutionary time scales, large ranges are more likely to be fragmented, given the variability in effectiveness of marine biogeographical barriers, increasing the chances of vicariance. Therefore, considering that large body sized herbivorous fishes are likely to have larger ranges, their rate of diversification through vicariance may be higher than small-medium body sized herbivorous species, for example through peripheral speciation. Please note, however, that this explanation provides a comparison within the body size range of herbivores only. To understand the comparison

with other trophic groups, it is important to connect the long-distance colonization process with the reef flat invasion explained throughout the discussion (Lines 281-297; and clarified in comment #6).

Comment#11 (R#2): The link with a Miocene diversification event seems weak – is there at least a way to illustrate the timing of diversification in line with this event? In addition, it is not made clear in this manuscript why reef rearrangement in Miocene happened or why this should influence fish diversification. The Miocene is also the time of onset of polar glaciation and increased latitudinal diversity gradients, and may be associated with a decline in diversification in the tropics (Rabosky et al., 2018). Thus, it seems a less than obvious time for reef fish diversification to increase.

Author's reply: Thank you for this suggestion, we have now produced a figure that illustrates the timing of diversification of reef fish lineages per trophic group in the last 100 Myr (supplementary fig. S5). From this lineage through time plot, it becomes evident that diversification increased during the Miocene, particularly in herbivores/detritivores, planktivores and sessile invertivores. These three trophic groups show higher slopes in the relationship between number of lineages and time during the Miocene when compared to mobile invertivores, generalized carnivores and omnivores. By contrast, these other trophic groups show a steadier diversification since the Eocene-Oligocene. A reference to this figure is now provided in the results section (Line 198).

In addition to this figure, we also added a new paragraph in the introduction that clarifies the influence of Miocene reef rearrangements in fish diversification (Lines 69-82). In essence, there is a vast literature identifying the Miocene as the most important phase for reef fish diversification (Bellwood & Wainwright, 2002; Renema *et al.*, 2008; Cowman *et al.*, 2009; Cowman & Bellwood, 2011; Bellwood *et al.*, 2012; Cowman & Bellwood, 2013; Bellwood *et al.*, 2015; Dornburg *et al.*, 2015; Leprieur *et al.*, 2016; Bellwood *et al.*, 2017). Unfortunately, the word limitation for short format papers does not allow us to explain in detail the multitude of factors influencing reef fish diversification in the Miocene. However, a recent review led by one of the authors of the present manuscript thoroughly describes the process for both reef fishes and corals (Bellwood *et al.* 2017 – Section (3) Phase 5: a modern coral reef emerges (34–5.3 Ma)). To summarise, the Miocene was a period marked by major

geomorphological changes in marine environments that resulted in the formation of the Indo-Australian-Archipelago (IAA) biodiversity hotspot. This process likely fostered diversification of reef fish lineages, considering the opportunities for vicariance and range expansion promoted by the geographical complexity of the IAA. In addition to these geological processes, the Miocene was a period in which reef fishes: (i) occupied new habitats; (ii) established new interactions with corals; and (iii) experienced trophic innovations. This summarized version of events is now provided in the new paragraph of the introduction, providing a clearer temporal link between reef rearrangements and our results.

Comment#12 (R#2): -288: Why are extinction rates slightly higher for omnivores? Are they really extinction rates if omnivory is transitional between other states, or is just that such lineages don't exist in that space for long? Line 308 says estimating extinction rates without fossils is problematic for small fishes and gobies but MuSSE was the only analysis applied regardless. Line 341 points out omnivory is not evolutionarily stable.

Author's reply: This is a good comment. As mentioned in the response to comment #3, we have now restructured our paragraph that discusses the Omnivores and removed the section highlighting higher extinction rates. Even though MuSSE results provide estimates of extinction rates per trophic group and our sample size minimizes potential problems associated with it (Beaulieu & O'Meara, 2015), we have followed your suggestion to not put weight on those estimates in our discussion. This is because we agree with your observation that transition rates might be part of the explanation. Thus, we included a sentence in the paragraph stating this idea (Lines 332-334). Finally, it is important to clarify that our note of caution for small fishes in the discussion is just a recommendation for future studies to also include fossils if their sole objective is to estimate extinction rates. This would be a better approach than relying exclusively on molecular phylogenies.

Comment#13 (R#2): 359: Could other factors be sociality and sexual selection, given that the herbivorous fishes tend to live in large groups?

Author's reply: Sociality and sexual selection are indeed interesting factors that have not been evaluated in our study but could potentially have influenced reef fish diversification patterns. However, we believe that they are not necessarily related to our trophic results for

a set of reasons. First, herbivores have considerable variability in sociality, from lineages commonly forming large groups to others forming only pairs (e.g. most rabbitfishes and *Zebrasoma* surgeonfishes, Brandl & Bellwood, 2013). Second, there are multiple non-herbivorous fish families that also form large groups but have low diversification rates based on our results (e.g. haemulids and holocentrids). So far, evidence suggests that traits associated with sexual selection might have been important in driving very recent (i.e. last 3 Myr) patterns of speciation that seems, thus, to be independent of trophic identity (e.g. Choat *et al.*, 2012 – parrotfishes; Salis *et al.*, 2018 – clownfishes; Hemingson *et al.*, 2019 – butterflyfishes; Hench *et al.*, 2019 – hamlets). Therefore, we believe that sociality and sexual selection should be analysed independently in future efforts to explain finer-scale patterns of reef fish diversification. We appreciate your insight.

Comment#14 (R#2): 363: Evidence for herbivores triggering Miocene reef formation seems circumstantial. This needs more information on patterns of reef building. What about Eocene reef-building? Early herbivores are found at places like Monte Bolca, where reef fishes exhibit more disparity than modern fishes (see papers). Couldn't this result come from the use of living fishes alone, representing a turnover event rather than initial reef-building?

Author's reply: Indeed, surgeonfishes and rabbitfishes have been described from the Eocene records of Monte Bolca, and their generic diversity was higher at that point in time when compared to extant genera. However, we would like to point out that this generic diversity in the Eocene was not necessarily reflected in higher morphological disparity. Firstly, there is no fossil evidence of parrotfishes (Scarini) in the Eocene, and they likely arose later in the Oligocene-Miocene (Siqueira *et al.*, 2019). Secondly, as noted by Bellwood *et al.*, (2014): "Although Eocene surgeonfishes likely played a critical role as herbivores during the origins of modern coral reefs, they lacked the novel morphologies seen in modern *Acanthurus* and *Siganus* (including eyes positioned high above their low-set mouths)". This is critical for understanding some of the processes explained in our manuscript, because these modern morphologies arose during the Miocene and are associated with exposed, high-turnover shallow reef habitats. Therefore, these morphological novelties in the Miocene mark the colonization of the reef flat by fishes (Bellwood *et al.* 2014), which is discussed in our manuscript (Lines 275-280).

Regarding Eocene reef-building, as Bellwood *et al.* (2017) notes: "Coral reefs as we know them today may have been rare, if they existed at all, in the shallow waters of the early Cenozoic. Indeed, in their description of some of the earliest *Acropora* fossils, Wallace & Rosen (2006) suggest that these early corals grew on patch reefs similar to those found today in Moreton Bay, Australia; a marginal high-sediment system with small coral-bearing outcrops. This patch-reef configuration appears to have been widespread in the Paleocene and Eocene (Zamagni *et al.*, 2012)". The paleontological evidence unequivocally shows an expansion of carbonate platforms in the Indo-Australian-Archipelago and an increased diversification of scleractinian corals in the Miocene (e.g. Wallace & Rosen, 2006; Mihaljević *et al.*, 2014; Santodomingo *et al.*, 2015). Although some herbivorous reef fishes were present in the Eocene, they did not seem to have had the morphologies to influence reef formation at that time since reefs had a very different configuration. We agree that molecular phylogenies might bias the precise origin of specific morphologies or taxonomic groups. However, in this case, the temporal evidence for the onset of scleractinian diversification comes from the fossil record, which is unlikely to suffer from the same biases. To help clarify this point, we now provide more information on patterns of Eocene reef building in our discussion (Lines 285-288). Thank you for pointing out this source of potential confusion.

Comment#15 (R#2): Reef fish phylogeny: Wouldn't it have been better to use data from FishBase to prune the tree to contain only specific reef-associated taxa rather than entire families that are at least 20% reef-associated (which seems really low when you are test reef-associated diversification, it seems like most coastal fishes would end up included)? It seems like this approach is confounded by diversification patterns shaped by the conditions of alternative habitats, or the influence of transients. This would have produced a smaller tree but possibly stronger results.

Author's reply: As noted in our response to comment #2, pruning the tree to contain only specific reef-associated taxa would likely result in biased estimates of diversification rates. The effect of incomplete sampling on estimates of diversification was recently reported by Chang *et al.* (2019). Thus, it was important to include all members of families containing at least 20% of reef-associated taxa in our sampling to reach realistic estimates of diversification rates. Although we agree that this means having non-reef taxa included in our

study, we found strong evidence that it has not confounded the diversification patterns observed. Indeed, when we ran the analysis considering only the "consensus" reef fish families (see comment #18) the patterns were the same but with a slight increase in support (model precision) and in the importance of trophic identity (results Lines 176-181; supplementary fig. 2). Having these two complementary approaches in the study was important as it enabled us to conclude that the diversification results were most strongly associated with the history of coral reefs and not with peripheral environments that also support 'reef-associated' fish species (discussion Lines 406-408).

Comment#16 (R#2): It also seems like the tree should have been recalibrated at a lower level, since the original tree contained 139 fossils for all ray-fins. The first occurrences of specific genera would have allowed for better estimation of diversification rates considering the event of interest (Miocene reef building) is only 8 million years out. How do you know your divergence times are realistic for this subset?

Author's reply: As noted in our response to comment #5, although the tree from Rabosky *et al.* (2018) contained all ray-finned fishes, the fossils used for calibrating their tree encompassed a large time stratum that includes very recent dates. Out of the 139 fossils used, 42 had minimum lower bounds that lie within the Neogene, which comprises our main period of interest (last 23 million years). In addition, we would not be able to add many more fossils than the ones already used in their calibration scheme given that fossils of reef fishes belonging to extant genera are rare (see Bellwood *et al.*, 2019 for examples in labrids, one of the most speciose families of reef fishes). Finally, we know that the divergence times from our backbone tree are realistic for the subset of reef-associated fishes because they largely agree with the crown ages of multiple important family-level phylogenies that have been previously estimated using Bayesian methods.

Comment#17 (R#2): 470: The methods in section d need more explanation in text for why this machine learning approach is preferred over prior or other existing modelling approaches, rather than a reference to a different paper followed by parameter selection criteria.

Author's reply: We have now added a more detailed explanation of the advantages of the machine learning approach in our methods section (Lines 533-536).

Comment#18 (R#2): 505: What is a "consensus family"?

Author's reply: The concept of "consensus" coral reef fish families was first defined by Bellwood (1996), who established a list of all families that would be found on a coral reef irrespective of its biogeographic location (i.e. Acanthuridae, Apogonidae, Blenniidae, Carangidae, Chaetodontidae, Holocentridae, Labridae, Mullidae and Pomacentridae). After that, Bellwood & Wainwright (2002) expanded the list to include those taxa that are found on, and are characteristic of, coral reefs (i.e. the consensus list plus taxa characteristic of reefs in specific areas). Therefore, in our study, we considered the consensus list plus the families Pomacanthidae and Serranidae as our definition of families universally occurring in coral reefs, rather than 'reefs' *sensu lato*. This definition is given in the results section (Lines 176-177). Additionally, we now provide the list of families in the methods section (Lines 569-571) for clarity.

Comment#19 (R#2): 516: Extinction estimated using MuSSE. What about the issues with estimating extinction in trees containing only living taxa? Same with diversification rates.

Author's reply: Although there are issues with estimating extinction rates from molecular phylogenies, the State Speciation and Extinction (SSE) models are still quite robust for detecting historical patterns of character evolution. Additionally, it has already been reported that larger sample sizes minimize the issues associated with extinction (and consequently diversification) rate estimates from phylogenies containing only living taxa (Beaulieu & O'Meara, 2015). Therefore, considering that we are working with near complete trees, our results provide the best that can currently be estimated from phylogenies built with extant taxa in terms of diversification rates.

Comment#20 (R#2): 540: Which function was used in Phytools for stochastic character mapping?

Author's reply: The stochastic character mappings were performed with the *make.simmap* function from the '*phytools*' R package. We added this information in the methods section (Lines 616-617) for clarity. Thank you for your care in checking our manuscript.

Authors' replies to the comments made by Reviewer #3

Comment#1 (R#3): This paper aims to explore the relative importance of ecological and geographical factors explaining heterogeneity in the tempo of lineage diversification among major reef-associated fish families. Along this work, the authors applied cutting-edge phylogenetic comparative methods on near complete phylogenetic trees retrieved from Rabosky et al. (2018). At the first glance, it is rather intuitive that feeding habits would be a major driver of diversification in the hyper diverse ichthyofauna living in reef ecosystems. So, I am not really surprised by the main results of the present study. However, even if many ichthyologists likely share my opinion, the authors made a good job by providing strong supports on this hypothesis at a very large taxonomic fish scale. Section (b) of results (lines 94-158) are probably the most satisfying ones of the present MS but I am much more concerned about the section (c) of results (lines 160-197). Accordingly, I have listed some comments that should be addressed prior to an acceptance for publication.

Author's reply: Thank you for your comments, we appreciate your positive comments and help in improving the manuscript. Although it has been previously suggested that feeding habits might drive diversification rate patterns in some reef fish groups, our study is the first to demonstrate this on a large phylogenetic scale, as you pointed out. More importantly, we show that the influence of trophic identity on diversification rates is much stronger than all the other ecological or geographical factors analysed, which is a very novel factor of the present study. In this revised version, we have addressed all your comments, that have helped considerably in improving the manuscript. Please, find below our detailed responses with line numbers referring to the revised version with inline track changes. It is our hope that you find this improved version suitable for publication.

Comment#2 (R#3): The introductory part is broadly oriented, mainly suggesting that various biotic and abiotic factors could sustain variation in the tempo of reef fish lineage diversification. To my opinion, the authors should work better to introduce (their) expectations about the impact of feeding novelties and size variation on lineage diversification. Various studies already showed

that trophic attributes acted as a major driving force during reef-associated fish evolution (e.g. Price et al. 2010, Frédérich et al. 2013, Friedman et al. 2016, Borstein et al. 2019). I encourage the authors to use these studies to build some assumptions about the link between trophic evolution and diversification (taxonomic, morphological and functional diversity) in reef fishes.

Author's reply: We have now restructured our introduction by adding a new paragraph that highlights the importance of trophic innovations as a potential factor influencing reef fish diversification rates (Lines 69-82), adding some of the suggested references. We believe that this new introduction better orientates the reader to the factors we analysed in the study.

Comment#3 (R#3): Results related to figures 1, 2 & 3 are the strength of the present paper. On the other hand, the authors failed to connect their results from figures 4 & 5 to a recent paper published in Ecology Letters by Gajdzik et al. (2019). Even if Gajdzik and colleagues only studied Labridae and Pomacentridae (in comparison to the larger sampling of fish families of the present study), they already studied variation in the tempo of lineage diversification among trophic guilds. They also illustrated the pivotal role of a generalist feeding strategy during the evolutionary history of reef-associated fishes. This « oversight » is detrimental. Indeed, when the authors will include the work of Gajdzik et al. (2019) in their list of references and their considerations, 40% of the results from the present MS will not appear as « new » as expected. The miss of such a key paper does not fit the standard of bibliographic works required for scientific research in general and the expectations from a journal like Nature Communications. I urge the authors to include the work of Gajdzik et al. (2019) in their paper and re-work their introduction, results and discussion accordingly.

Author's reply: Thank you for suggesting this relevant reference. As we noted in the response to comment #4 by reviewer #1, by focusing on large-scale results we may not have paid sufficient attention to the details of previous studies that worked on selected reef fish families. Indeed, some of the results found for Pomacentridae and Labridae in Gajdzik *et al.* (2019) are complementary to our results found for the full reef fish tree. Therefore, we have now included this reference in multiple parts of our manuscript (Lines 84-86; 230-231; 337-340; 382-384; 387-391) to highlight the similar findings. We would like to point out, however, that the similarities that the reviewer suggests are mostly limited to our results on transitions between trophic groups. Even though Gajdzik *et al.* (2019) analysed lineage diversification

among trophic guilds, their scheme of trophic classification differs from ours in the sense that they considered habitat use (e.g. benthic versus pelagic) in combination with diet to classify their species. Our classification scheme was based solely on diets. This, plus a 10-fold increase in species considered, limits our ability to make direct comparisons. As you correctly pointed out, our main results are presented in figures 1, 2 and 3, where we employ a framework that has not been applied in any previous studies to our knowledge. Hence, given these striking distinctions in terms of species and analytical breadth, we see our paper and that of Gajdzik *et al.* (2019) as valuable complementary papers each addressing patterns at different scales.

Comment#4 (R#3): Page 4, line 81: Define « net diversification rate » (= speciation - extinction) for its first reference in the text.

Author's reply: Defined as suggested (Line 101).

Comment#5 (R#3): Page 7, lines 125-126: How do the authors identify three size categories? This appears really unclear to me.

Author's reply: This division was based on the distribution of body sizes within our dataset in a way that each size category (small, medium and large) had roughly similar number of species. We have modified the highlighted sentence to better explain this division and to clarify this point (Lines 152-154).

Comment#6 (R#3): Page 7, line 129: The authors should be cautious when using terms like « specialization », « specialized »,… How a group made of detritivorous, macroalgivorous and filamentous algivorous species would be more specialized than groups made of omnivorous or carnivorous species?? Definition of specialization can be tricky and the authors' definition of specialization have to be clearly stated.

Author's reply: This is a most helpful comment and, as this aspect was not a primary focus of the study, the potential for confusion means that the manuscript would be much clearer if it was removed. We agree that the binary division between 'specialized' and 'generalized' trophic groups would require clearer a definition, which was lacking in the first version of

our manuscript. Moreover, we agree that such division can be problematic. Therefore, to follow this helpful suggestion and the comment #3 by Reviewer #1, we have now removed all uses of the terms within the manuscript. Thank you for pointing this out.

Comment#7 (R#3): Page 12, lines 200-202: Many other references could be added here. Please, avoid self-citations and add, for example, some of the references cited above (and other papers).

Author's reply: We have added other relevant citations in the suggested sentence (Lines 229-231), and elsewhere.

Comment#8 (R#3): Page 12, lines 210-215: See results from Gajdzik et al. (2019) and include them in your discussion.

Author's reply: The results from Gajdzik *et al.* (2019) have now been discussed in multiple parts of our manuscript (Lines 84-86; 230-231; 337-340; 382-384; 387-391).

Comment#9 (R#3): Page 13, lines 245-256: Please, provide more explanations on why the expansion of corals in shallow waters may sustain the diversification of sessile invertivores and planktivores. How does water flow vary during/after the rise of reef flat?

Author's reply: We now provide more details about the potential relationship between the diversification of sessile invertivores and planktivores, and the expansion of corals in shallow waters (Lines 288-297). In the case of sessile invertivores, this relationship can be simply explained by the increase in the range of available food sources in shallow waters. In planktivores, on the other hand, higher diversification can be linked to the refuge provided by corals against predators and water flow in highly productive and highly hydrodynamic shallow reef environments, where food availability might be increased. Please note that we did not claim that water flow has changed after or during the rise of the reef flat, we only associated the increased topographical complexity provided by corals with increased sheltering opportunities for planktivores against high water flow and predators.

Comment#10 (R#3): Page 14, lines 257-282: Discussion about natural selection on large body sized herbivores is interesting but highly speculative. The authors should shorten this section of

the discussion but they should suggest some tips for testing this in next works. As an example, models of size evolution (BM - Brownian motion and multiple OU - Ornstein Uhlenbeck models) could be fitted and compared in order to assess an optimum for large sized grazing fishes on reef flat habitat.

Author's reply: Thank you for this suggestion, we have shortened the discussion about predation and large body sized herbivores. Additionally, we have modified that paragraph to include recommendations for future studies to examine models of size evolution within herbivorous fishes (Lines 312-314), as suggested.

Comment#11 (R#3): Pages 15-17: All this part of the discussion should be re-worked by including the results from Gajdzik et al. (2019).

Author's reply: We re-worked the suggested part of the discussion to include the findings of Gajdzik *et al.* (2019) (Lines 382-384; 387-391).

Comment#12 (R#3): Page 17, lines 323-326: This sentence is unclear. What does « departures from typical morphologies and behaviors towards planktivory » mean?

Author's reply: The referred sentence was suggesting that individual lineages that transition to planktivory present departures from the typical morphologies and behaviours of the family (see examples in Floeter *et al.*, 2018). However, since the sentence was unclear in the previous version, we now rephrased it, providing explicit examples of morphological modifications associated with transitions to planktivory (Lines 371-378).

Comment#13 (R#3): A great part of the results/discussion focus on the diversification of large herbivorous/detritivores fishes. However BAMM analyses also revealed high rates of lineage diversification in snappers (Lutjanidae) and butterflyfishes (Chaetodontidae). Then, this information is diluted in the rest of the text. How the high rates of diversification in both families are supported by MuSSE results? What can explain such high rates of net diversification in both families which include fish species feeding on animal prey?

Author's reply: The BAMM analysis did indeed reveal high diversification rates in Lutjnidae and Chaetodontidae. This is highlighted in our results section (Lines 103-106). Although

some species in Chaetodontidae do feed on mobile invertebrates, coral feeding, including obligate coral feeding, is also very common in *Chaetodon*, the most speciose genus within the family. Therefore, the results for this particular family do not depart from the general trend that relates trophic evolution with diversification rates. The results for Lutjanidae, however, represent a case in which diversification rates might be decoupled from the described pattern of trophic evolution. Hence, it is important to note that trophic groups do not explain all the variance found for diversification rates within our model. At best, it explains 40% of variance, with a substantial part still left for the myriad other factors that could affect diversification (i.e. geography, habitat or behavioural factors). This means that, even though trophic identity represents the most important factor in the model, a large amount of variance remains unexplained. In our 'model considerations' section of the discussion (Lines 397-408), we deal with some of these aspects. Therefore, the Chaetodontidae appear to substantially support the main conclusions of the MuSSE results (i.e. high diversification rates for sessile invertivores), while in the Lutjanidae, it substantially exceeds the average diversification of generalised carnivores, showing that this family likely responded to other factors, driving some of the high values within the distribution of this group. We have now modified our Results section (Lines 194-198) to clarify this point.

Comment#14 (R#3): Figures: why do the authors chose silhouette from different fish families for illustrating trophic strategies? It is not really appropriate. It is misleading because, first, we could primarily associated silhouettes to taxonomic groups (fish family). Moreover we know that one fish family generally includes more than one trophic strategy. So, please, modify this iconography.

Author's reply: We tried alternative icons but found that they caused issues with clarity. We therefore checked to ensure that the silhouettes were of taxa that are quite characteristic of each trophic group. This iconography is particularly appealing considering the broad readership of *Nature Communications*, who may be unfamiliar with the variation within many reef fish families.

References

Barneche, D.R., Robertson, D.R., White, C.R. & Marshall, D.J. (2018) Fish reproductive-energy output increases disproportionately with body size. *Science*, **360**, 642–645.

Beaulieu, J.M. & O'Meara, B.C. (2015) Extinction can be estimated from mederately sized molecular phylogenies. *Evolution*, **69**, 1036–1043.

Bellwood, D.R. (1996) The Eocene fishes of Monte Bolca: the earliest coral reef fish assemblage. *Coral Reefs*, **15**, 11–19.

Bellwood, D.R., Goatley, C.H.R. & Bellwood, O. (2017) The evolution of fishes and corals on reefs: form, function and interdependence. *Biological Reviews*, **92**, 878–901.

Bellwood, D.R., Goatley, C.H.R., Brandl, S.J. & Bellwood, O. (2014) Fifty million years of herbivory on coral reefs: fossils, fish and functional innovations. *Proceedings of the Royal Society B: Biological Sciences*, **281**, 20133046.

Bellwood, D.R., Goatley, C.H.R., Cowman, P.F., Bellwood, O. & Mora, C. (2015) *The evolution of fishes on coral reefs: fossils, phylogenies and functions*. *Ecology of Fishes on Coral Reefs* (ed. by C. Mora), p. 388. Cambridge University Press, Cambridge.

Bellwood, D.R., Renema, W. & Rosen, B.R. (2012) *Biodiversity hotspots, evolution and coral reef biogeography*. *Biotic Evolution and Environmental Change in Southeast Asia* (ed. by D.J. Gower), K. Johnson), J. Richardson), B. Rosen), L. Rüber), and S. Williams), pp. 2–32. Cambridge University Press, Cambridge.

Bellwood, D.R., Schultz, O., Siqueira, A.C. & Cowman, P.F. (2019) A review of the fossil record of the Labridae. *Annalen des Naturhistorischen Museums in Wien, Serie A*, **121**, 125–193.

Bellwood, D.R. & Wainwright, P.C. (2002) *The history and biogeography of fishes on coral reefs*. *Coral reef fishes: dynamics and diversity on a complex ecosystem* (ed. by P.F. Sale), pp. 5–32. Academic Press, San Diego.

Brandl, S.J. & Bellwood, D.R. (2013) Morphology, sociality, and ecology: can morphology predict pairing behavior in coral reef fishes? *Coral Reefs*, **32**, 835–846.

Chang, J., Rabosky, D.L. & Alfaro, M.E. (2019) Estimating diversification rates on incompletely sampled phylogenies: theoretical concerns and practical solutions. *Systematic Biology*, **0**, 1–10.

Choat, J.H. & Axe, L.M. (1996) Growth and longevity in acanthurid fishes; An analysis of

otolith increments. *Marine Ecology Progress Series*, **134**, 15–26.

Choat, J.H., Klanten, O.S., Van Herwerden, L., Robertson, R.D. & Clements, K.D. (2012) Patterns and processes in the evolutionary history of parrotfishes (Family Labridae). *Biological Journal of the Linnean Society*, **107**, 529–557.

Cowman, P.F. & Bellwood, D.R. (2011) Coral reefs as drivers of cladogenesis: expanding coral reefs, cryptic extinction events, and the development of biodiversity hotspots. *Journal of Evolutionary Biology*, **24**, 2543–2562.

Cowman, P.F. & Bellwood, D.R. (2013) The historical biogeography of coral reef fishes: global patterns of origination and dispersal. *Journal of Biogeography*, **40**, 209–224.

Cowman, P.F., Bellwood, D.R. & van Herwerden, L. (2009) Dating the evolutionary origins of wrasse lineages (Labridae) and the rise of trophic novelty on coral reefs. *Molecular phylogenetics and evolution*, **52**, 621–631.

DeCarlo, T.M. & Harrison, H.B. (2019) An enigmatic decoupling between heat stress and coral bleaching on the Great Barrier Reef. *PeerJ*, **2019**, 1–30.

Dibattista, J.D., Roberts, M.B., Bouwmeester, J., Bowen, B.W., Coker, D.J., Lozano-Cortés, D.F., Howard Choat, J., Gaither, M.R., Hobbs, J.P.A., Khalil, M.T., Kochzius, M., Myers, R.F., Paulay, G., Robitzch, V.S.N., Saenz-Agudelo, P., Salas, E., Sinclair-Taylor, T.H., Toonen, R.J., Westneat, M.W., Williams, S.T. & Berumen, M.L. (2016) A review of contemporary patterns of endemism for shallow water reef fauna in the Red Sea. *Journal of Biogeography*, **43**, 423–439.

Dornburg, A., Moore, J., Beaulieu, J.M., Eytan, R.I. & Near, T.J. (2015) The impact of shifts in marine biodiversity hotspots on patterns of range evolution: evidence from the Holocentridae (squirrelfishes and soldierfishes). *Evolution*, **69**, 146–161.

Floeter, S.R., Bender, M.G., Siqueira, A.C. & Cowman, P.F. (2018) Phylogenetic perspectives on reef fish functional traits. *Biological Reviews*, **93**, 131–151.

Gajdzik, L., Aguilar-Medrano, R. & Frédérich, B. (2019) Diversification and functional evolution of reef fish feeding guilds. *Ecology Letters*, **22**, 572–582.

Hemingson, C.R., Cowman, P.F., Hodge, J.R. & Bellwood, D.R. (2019) Colour pattern divergence in reef fish species is rapid and driven by both range overlap and symmetry. *Ecology Letters*, **22**, 190–199.

Hench, K., Vargas, M., Höppner, M.P., McMillan, W.O. & Puebla, O. (2019) Inter-chromosomal

coupling between vision and pigmentation genes during genomic divergence. *Nature Ecology and Evolution*, **3**, 657–667.

Horn, M.H. (1989) Biology of marine herbivorous fishes. *Oceanography and Marine Biology Annual Reviews*, **27**, 167–272.

Leprieur, F., Descombes, P., Gaboriau, T., Cowman, P.F. & Parravicini, V. (2016) Plate tectonics drive tropical reef biodiversity dynamics. *Nature Communications*, **7**, 11461.

Lobato, F.L., Barneche, D.R., Siqueira, A.C., Liedke, A.M.R., Lindner, A., Pie, M.R., Bellwood, D.R. & Floeter, S.R. (2014) Diet and diversification in the evolution of coral reef fishes. *PLoS ONE*, **9**, e102094.

Luiz, O.J., Allen, A.P., Robertson, D.R., Floeter, S.R., Kulbicki, M., Vigliola, L., Becheler, R. & Madin, J.S. (2013) Adult and larval traits as determinants of geographic range size among tropical reef fishes. *Proceedings of the National Academy of Sciences of the United States of America*, **110**, 16498–16502.

Mihaljević, M., Renema, W., Welsh, K. & Pandolfi, J.M. (2014) Eocene-Miocene Shallow-Water Carbonate Platforms and Increased Habitat Diversity in Sarawak, Malaysia. *Palaios*, **29**, 378–391.

Morais, R.A. & Bellwood, D.R. (2018) Global drivers of reef fish growth. *Fish and Fisheries*, **19**, 874–889.

Mouillot, D., Villéger, S., Parravicini, V., Kulbicki, M., Arias-Gonzáleze, J.E., Bender, M., Chabanet, P., Floeter, S.R., Friedlander, A., Vigliola, L. & Bellwood, D.R. (2014) Functional over-redundancy and high functional vulnerability in global fish faunas of tropical reefs. *PNAS*, **111**, 13757–13762.

Parravicini, V., Villéger, S., Mcclanahan, T.R., Arias-González, J.E., Bellwood, D.R., Belmaker, J., Chabanet, P., Floeter, S.R., Friedlander, A.M., Guilhaumon, F., Vigliola, L., Kulbicki, M. & Mouillot, D. (2014) Global mismatch between species richness and vulnerability of reef fish assemblages. *Ecology Letters*, **17**, 1101–1110.

Rabosky, D.L., Chang, J., Title, P.O., Cowman, P.F., Sallan, L., Friedman, M., Kaschner, K., Garilao, C., Near, T.J., Coll, M. & Alfaro, M.E. (2018) An inverse latitudinal gradient in speciation rate for marine fishes. *Nature*, **559**, 392–395.

Renema, W., Bellwood, D.R. & Braga, J.C. (2008) Hopping hotspots: global shifts in marine biodiversity. *Science*, **321**, 654–657.

Salis, P., Roux, N., Soulat, O., Lecchini, D., Laudet, V. & Frédérich, B. (2018) Ontogenetic and phylogenetic simplification during white stripe evolution in clownfishes. *BMC Biology*, **16**, 90.

Santodomingo, N., Wallace, C.C. & Johnson, K.G. (2015) Fossils reveal a high diversity of the staghorn coral genera Acropora and Isopora (Scleractinia: Acroporidae) in the Neogene of Indonesia. *Zoological Journal of the Linnean Society*, **175**, 677–763.

Siqueira, A.C., Bellwood, D.R. & Cowman, P.F. (2019) Historical biogeography of herbivorous coral reef fishes: The formation of an Atlantic fauna. *Journal of Biogeography*, **46**, 1611–1624.

Siqueira, A.C., Oliveira-santos, L.G.R., Cowman, P.F. & Floeter, S.R. (2016) Evolutionary processes underlying latitudinal differences in reef fish biodiversity. *Global Ecology and Biogeography*, **25**, 1466–1476.

Tyberghein, L., Verbruggen, H., Pauly, K., Troupin, C., Mineur, F. & De Clerck, O. (2012) Bio-ORACLE: A global environmental dataset for marine species distribution modelling. *Global Ecology and Biogeography*, **21**, 272–281.

Wainwright, P.C. (2007) Functional Versus Morphological Diversity in Macroevolution. *Annual Review of Ecology, Evolution, and Systematics*, **38**, 381–401.

Wallace, C.C. & Rosen, B.R. (2006) Diverse staghorn corals (Acropora) in high-latitude Eocene assemblages: Implications for the evolution of modern diversity patterns of reef corals. *Proceedings of the Royal Society B: Biological Sciences*, **273**, 975–982.

Zamagni, J., Mutti, M. & Košir, A. (2012) The evolution of mid Paleocene-early Eocene coral communities: how to survive during rapid global warming. *Palaeogeography, Palaeoclimatology, Palaeoecology*, **317–318**, 48–65.

Reviewers' comments:

Reviewer #3 (Remarks to the Author):

The authors have addressed all my previous comments and those from the two other reviewers.

Reviewer #4 (Remarks to the Author):

I enjoyed reading your paper on the drivers of reef fish diversification. I thought it was a particularly thorough and thoughtful contribution to the literature of this area that was both generally quite well done (though I have some concerns, see below) and well-described. I particularly appreciated all the interesting natural history (for lack of a better term) nuance that helped guide your interpretations. As I mentioned above, I do however have some concern regarding some of the phylogenetic methods.

Major comments:

1. Recently, my collaborator Stilianos Louca and I proved what many in the field might have suspected/argued: that speciation and extinction rates cannot be disentangled from phylogenetic data alone (Louca and Pennell BiorXiv DOI: 10.1101/719435; now in press at Nature). While others have, as you point out in the present paper, argued that extinction rates cannot be accurately estimated from molecular phylogenies, we make the more general case that, if rates are thought to vary through time, then neither can speciation rates, net diversification rates, or relative extinction rates. No matter how much data one has, one can never come up with a consistent estimate of any of these parameters. I am still not sure what to recommend following this result -- it came as a surprise to me as well -- but I do think that it is something that needs to be addressed at some length in this paper.

One reason you might be optimistic: while we shown that temporal trends in parameters are not identifiable from phylogenies of extant species, we proved one can reliably estimate very recent speciation rates. Since the DR statistic (and the BAMM tip-rates) you use mostly depend on information from the tips and not deeper in the tree where things are murkier, I think you could make the case that your results are likely to be robust to parameter non-identifiability. But as I said, I don't have all the answers here.

2. Setting that aside for a moment, I think the MuSSE/HiSSE analyses are rather redundant with the BAMM/DR analyses. Both are essentially asking the same question with different approaches/assumptions. Of course it is often a good idea to test the same hypothesis with multiple methods, and judging from the correspondence with previous reviewers, is something that you were explicitly asked to do. However, I think it feels a bit odd to me that you i) include both results in the main text (Figs 2b and 4 are essentially the same results); yet ii) don't try to unpack why these might tell you anything different. I would request that you either relegate one of the analyses (presumably the SSE analyses since you likely want to keep the boosted regression analyses) to the supp mat and just mention that your results were generally quite robust to how you assessed state dependent diversification or spend more time in the text discussing similarities and differences between the approaches. As it stands now, it is rather disorienting.

3. Given your results from the diversification analyses, conducting stochastic character mapping without including state-dependent diversification (which phytools does not) will give you biased estimates of transition rates. The reasoning behind this problem is best articulated by Maddison 2006 Evolution and was one of the original motivations for developing the BiSSE approach. As such I think that your two primary types of analyses (diversification and transition rate analyses) and internally inconsistent. Fortunately there is potentially a straightforward solution: do a stochastic character analyses conditional upon the data, and all of the inferred parameters from the SSE analyses. I say potentially since I know such an approach is already implement for bisse (see

diversitree::make.asr.stoch()) but I am not sure about the corresponding function for HiSSE. In any case, this is something that I think needs to be addressed.

Minor comments:

Line 175: I did find it rather notable that the young radiations were generally diversifying faster than older groups as this appears to be a pattern that is widespread across many groups (including previous studies of fish diversification; Rabosky et al 2018). My co-authors and I put together a meta-analysis last year that bore this out even across a “random” selection of phylogenetic trees from the literature (Henao Diaz et al. 2019 PNAS). I am still unsure what precisely is causing this general pattern - and am always interested in ideas - but I do think it is worth pointing out that this result likely isn’t specific to fish.

Hope this review was helpful,
Matt Pennell

Authors' replies to the comments made by Reviewer #4

Comment#1 (R#4): I enjoyed reading your paper on the drivers of reef fish diversification. I thought it was a particularly thorough and thoughtful contribution to the literature of this area that was both generally quite well done (though I have some concerns, see below) and well-described. I particularly appreciated all the interesting natural history (for lack of a better term) nuance that helped guide your interpretations. As I mentioned above, I do however have some concern regarding some of the phylogenetic methods.

Author's reply: Dear Dr Pennell, thank you for the positive comments and constructive review of our manuscript, we were deeply encouraged by your feedback. We have addressed all of your points, modifying the manuscript accordingly. Please, find below more detailed responses explaining how we dealt with each comment. Note that line numbers refer to the revised version of the manuscript with inline track changes.

Comment#2 (R#4): Recently, my collaborator Stilianos Louca and I proved what many in the field might have suspected/argued: that speciation and extinction rates cannot be disentangled from phylogenetic data alone (Louca and Pennell BiorXiv DOI: 10.1101/719435; now in press at Nature). While others have, as you point out in the present paper, argued that extinction rates cannot be accurately estimated from molecular phylogenies, we make the more general case that, if rates are thought to vary through time, then neither can speciation rates, net diversification rates, or relative extinction rates. No matter how much data one has, one can never come up with a consistent estimate of any of these parameters. I am still not sure what to recommend following this result -- it came as a surprise to me as well -- but I do think that it is something that needs to be addressed at some length in this paper.

One reason you might be optimistic: while we shown that temporal trends in parameters are not identifiable from phylogenies of extant species, we proved one can reliably estimate very recent speciation rates. Since the DR statistic (and the BAMM tip-rates) you use mostly depend on information from the tips and not deeper in the tree where things are murkier, I think you could make the case that your results are likely to be robust to parameter non-identifiability. But as I said, I don't have all the answers here.

Author's reply: Thank you for bringing this key reference to our attention. Your paper represents a much needed contribution to the field of phylogenetic comparative methods by exposing an issue that needs to be addressed by all studies analyzing diversification dynamics with extant species phylogenies. Fortunately, as you pointed out, our study focuses mainly on recent estimates of diversification and should be robust to the non-identifiability issues described. Therefore, to comply with your suggestion, we have expanded our caveats section in the discussion (Lines 371-375) and the methods section (Lines 459-461), recognizing this potential issue and highlighting how our results may be relatively robust when confronting these important issues.

Comment#3 (R#4): Setting that aside for a moment, I think the MuSSE/HiSSE analyses are rather redundant with the BAMM/DR analyses. Both are essentially asking the same question with different approaches/assumptions. Of course it is often a good idea to test the same hypothesis with multiple methods, and judging from the correspondence with previous reviewers, is something that you were explicitly asked to do. However, I think it feels a bit odd to me that you i) include both results in the main text (Figs 2b and 4 are essentially the same results); yet ii) don't try to unpack why these might tell you anything different. I would request that you either relegate one of the analyses (presumably the SSE analyses since you likely want to keep the boosted regression analyses) to the supp mat and just mention that your results were generally quite robust to how you assessed state dependent diversification or spend more time in the text discussing similarities and differences between the approaches. As it stands now, it is rather disorienting.

Author's reply: Thank you for this thoughtful and helpful comment. We agree that the BAMM/DR analyses show very similar results to the MuSSE/HiSSE analyses, however, we believe that, in the context of our study, they are complementary rather than being redundant. Whereas the estimates from BAMM/DR were used to scrutinize the predictors of recent (tip) diversification rates independently of trait evolution, the MuSSE/HiSSE analyses were specifically used to investigate trait-dependent patterns of evolution throughout the whole structure of the tree (Methods section - Lines 577-579). Hence, this latter investigation provides a broader historical perspective of the evolution of trophic groups among reef fishes. Following your suggestion (option 2 above), we have expanded

multiple parts of the manuscript (Lines 83-85; 176-179; 306-308; 557-559), making this distinction between approaches clearer to the readers. We also include a paragraph in the discussion that provides possible explanations for the differences found between the two approaches (Lines 306-317).

Comment#4 (R#4): Given your results from the diversification analyses, conducting stochastic character mapping without including state-dependent diversification (which phytools does not) will give you biased estimates of transition rates. The reasoning behind this problem is best articulated by Maddison 2006 Evolution and was one of the original motivations for developing the BiSSE approach. As such I think that your two primary types of analyses (diversification and transition rate analyses) and internally inconsistent. Fortunately there is potentially a straightforward solution: do a stochastic character analyses conditional upon the data, and all of the inferred parameters from the SSE analyses. I say potentially since I know such an approach is already implement for bisse (see diversitree::make.asr.stoch()) but I am not sure about the corresponding function for HiSSE. In any case, this is something that I think needs to be addressed.

Author's reply: This is a good point, thank you for spotting this inconsistency. We agree that the stochastic character mapping analysis should have been performed considering state-dependent diversification. Therefore, we have now reframed the analysis considering the results from MuSSE, instead of using the default parametrization from *phytools*. Essentially, we have customized the 'make.simmap' function to perform stochastic mappings considering the results from ancestral state reconstructions based on the MuSSE model ('asr.marginal' function from *diversitree*). The details of this analysis are now provided in the methods section (Lines 594-600), and the customized function will be provided along with the code needed to reproduce the results pending acceptance. We would like to highlight that the results were very similar to those presented in the previous version of our manuscript (new Fig. 5), which suggests that rate heterogeneity had very little influence on the absolute number of transitions between reef fish trophic groups.

Comment#5 (R#4): Line 175: I did find it rather notable that the young radiations were generally diversifying faster than older groups as this appears to be a pattern that is widespread across many

groups (including previous studies of fish diversification; Rabosky et al 2018). My co-authors and I put together a meta-analysis last year that bore this out even across a "random" selection of phylogenetic trees from the literature (Henao Diaz et al. 2019 PNAS). I am still unsure what precisely is causing this general pattern - and am always interested in ideas - but I do think it is worth pointing out that this result likely isn't specific to fish.

Author's reply: Our results indeed corroborate the general pattern described by Henao Diaz *et al.* (2019), thank you for suggesting this reference. We have now modified a sentence in our discussion (Lines 244-246) to acknowledge that highest diversification rates within the last 20 Myr found for reef fishes reflect a general pattern across the tree of life. While this still needs to be investigated with other taxa, our results for reef fishes show that trophic innovations might be an important factor underpinning these changes on coral reefs.

REVIEWERS' COMMENTS:

Reviewer #4 (Remarks to the Author):

Thank you for addressing my comments in the previous round of reviews. I think the revised version of the manuscript is substantially improved and I am happy to recommend it for publication at this point. I sincerely think this is a very nice study and hope that it is well read and cited going forward.

Matt Pennell

Authors' replies to the comments made by Reviewer #4

Comment#1 (R#4): Thank you for addressing my comments in the previous round of reviews. I think the revised version of the manuscript is substantially improved and I am happy to recommend it for publication at this point. I sincerely think this is a very nice study and hope that it is well read and cited going forward. Matt Pennell.

Author's reply: Dear Dr Pennell, thank you for your valuable comments on the previous round of reviews and for recognizing our efforts to address them. We are grateful for your compliments and delighted that you enjoyed reading it.
